# Natural variation and gene regulatory basis for the responses of asparagus beans to soil drought

**DOI:** 10.3389/fpls.2015.00891

**Published:** 2015-10-27

**Authors:** Pei Xu, Menachem Moshelion, XiaoHua Wu, Ofer Halperin, BaoGen Wang, Jie Luo, Rony Wallach, Xinyi Wu, Zhongfu Lu, Guojing Li

**Affiliations:** ^1^Institute of Vegetables, Zhejiang Academy of Agricultural SciencesHangzhou, China; ^2^State Key Lab Breeding Base for Sustainable Control of Plant Pest and Disease, Zhejiang Academy of Agricultural SciencesHangzhou, China; ^3^The Robert H. Smith Faculty of Agriculture, Food and Environment, The Robert H. Smith Institute of Plant Sciences and Genetics in Agriculture, The Hebrew University of JerusalemRehovot, Israel; ^4^Department of Soil and Water Sciences, The Robert H. Smith Faculty of Agriculture, Food and Environment, Hebrew University of JerusalemRehovot, Israel; ^5^Institute of Digital Agriculture, Zhejiang Academy of Agricultural SciencesHangzhou, China

**Keywords:** asparagus bean (*Vigna unguiculata* ssp. *sesquipedalis*), cowpea, drought, GWAS, microarray, natural variation, water relations

## Abstract

Asparagus bean (*Vigna unguiculata* ssp. *sesquipedalis*) is the Asian subspecies of cowpea, a drought-resistant legume crop native to Africa. In order to explore the genetic variation of drought responses in asparagus bean, we conducted multi-year phenotyping of drought resistance traits across the Chinese asparagus bean mini-core. The phenotypic distribution indicated that the ssp. *sesquipedalis* subgene pool has maintained high natural variation in drought responses despite known domestic bottleneck. Thirty-nine SNP loci were found to show an association with drought resistance via a genome-wide association study (GWAS). Whole-plant water relations were compared among four genotypes by lysimetric assay. Apparent genotypic differences in transpiration patterns and the critical soil water threshold in relation to dehydration avoidance were observed, indicating a delicate adaptive mechanism for each genotype to its own climate. Microarray gene expression analyses revealed that known drought resistance pathways such as the ABA and phosphate lipid signaling pathways are conserved between different genotypes, while differential regulation of certain aquaporin genes and hormonal genes may be important for the genotypic differences. Our results suggest that divergent sensitivity to soil water content is an important mechanism configuring the genotypic specific responses to water deficit. The SNP markers identified provide useful resources for marker-assisted breeding.

## Introduction

Cowpea (*Vigna unguiculata*) is an important grain, vegetable, and fodder legume crop around the world. In Africa, South America and the Mediterranean basin, ssp. *unguiculata*, also known as African cowpea, is the dominant cultivar group grown mostly for use as grain. In Asia, ssp. *sesquipedalis*, also commonly referred to as the “asparagus bean” or “yardlong” bean due to its long tender pods that are harvested immature as a vegetable, is the most widely cultivated (Timko et al., 2007). Cowpea has long been recognized as a drought-resistant species, even outperforming groundnut, sorghum and pearl millet (*Pennisetum americanum*) under severe soil drought conditions (Ewansiha and Singh, 2006). However, both the capacity of cowpea plants to withstand water deficits and their stress reactions vary significantly by genotype (Watanabe et al., 1997; Muchero et al., 2008).

Plant physiologists and geneticists/breeders have long focused on understanding the physiological and genetic basis, respectively, of drought adaptation in cowpea. Physiologically, turgor-controlled leaflet movement (Schakel and Hall, 1979), a high leaf water potential (Bates and Hall, 1982), and partially opened stomata (Cruz de Carvalho et al., 1998) have been suggested to be key features for achieving high drought resistance in the African cowpea, while genotypic variation at the level of dehydration avoidance is correlated with the level of drought resistance (Belko et al., 2012). Geneticists have developed simple but reliable protocols to assess the levels of drought resistance at a large scale, such as visual scoring of drought-induced premature senescence, wilt, loss of stem greenness, and scoring of grain yield components (Muchero et al., 2008; Agbicodo et al., 2009). Using bi-parental mapping populations or natural populations, several QTLs or individual SNP loci were identified to be associated with these traits (Agbicodo, 2009; Muchero et al., 2009b, 2013). Efforts to characterize the gene expression related to drought resistance vs. susceptibility phenotypes have identified dozens of drought-responsive genes in cowpea (Iuchi et al., 1996; D'Arcy-Lameta et al., 2006). Nevertheless, large gaps currently exist among the various types of knowledge.

Since dehydration avoidance (a whole-plant trait) is considered to be the primary physiological mechanism responsible for the genotypic differences in drought resistance levels in cowpea (Bates and Hall, 1982; Likoswe and Lawn, 2008), the importance of surveying cowpea plant behaviors at the whole-plant level is reinforced. From the perspective of whole-plant water management, cowpea plants can be classified as “type I” or “type II” (Mai-Kodomi et al., 1999). Type I plants maintain high turgor and stay green for a longer time by ceasing growth and conserving moisture in all tissues. In contrast, Type II responses involve plants mobilizing moisture from older leaves to sustain growth of young tissues, resulting in rapid senescence of unifoliates. Although such classification has been used by cowpea breeders to choose parental lines for specific breeding programs, the physiological basis especially whole-plant water status in relation to the genotypic differences remain largely unknown. A major reason is that precise and representative assessment of whole-plant water relations is notoriously difficult, due to the spatial variability of soil types, their properties, changing soil-moisture conditions and other ambient conditions. To specifically measure drought avoidance, weeks of continuous assays are routinely required, making traditional facilities/methods based on non-automated and visual inspection impractical. The recent introduction of an advanced lysimetric facility offers a potential solution to this problem (Wallach et al., 2010). This system is based on a gravimetric system with soil probes; computer-controlled experimental planning, execution, and monitoring; and data analysis modules, thus providing automatic and real-time measurements to compare plant performance under non-stressed and stressed conditions.

Unlike its African progenitor, asparagus bean was domesticated in Asia and has adapted well to the milder local climate (Timko et al., 2007). There are currently no published data quantifying the genotypic differences in drought resistance in this subspecies, nor is the impact of artificial selection for agronomic traits on drought resistance understood. In this study, a multidisciplinary characterization of the natural variation in drought responses was carried out across the Chinese asparagus bean germplasm to dissect the genetic, physiological, and gene regulatory basis underlying the adaptive plasticity of this crop to soil drought. Our main findings include the apparent genotypic differences in transpiration patterns, the critical soil water threshold, and gene regulatory patterns in relation to dehydration avoidance among different varieties. We propose that divergent sensitivity to soil water content may represent an important adaptive mechanism for each genotype to its own climate. Thirty-nine SNP markers showing an association with various drought resistance traits were identified, providing useful resources for marker-assisted breeding of elite drought resistant varieties. Last but not least, we wish to underscore the often-neglected differences in the concepts of physiological and agronomic drought resistances for breeders to take into account when making their breeding programs.

## Materials and methods

### Plant materials

The plant materials used in this study comprised the 95-accession Chinese asparagus bean mini-core collection. This population had been genotyped with 1127 genome-wide distributed SNPs, based on which both the population structure (Q) and the relative kinships (K) were inferred (Xu et al., 2012). Two subpopulations namely the subgroup SV (standard vegetable) and NSV (non-standard vegetable) were partitioned in this population, and most individual pairs are found to be not or only weakly related.

### Visual phenotyping

Plants for visual phenotyping were grown under natural light conditions in a greenhouse in Haining, China (30°32′N/120°41′E). Each experiment included three replicates of drought-stressed plants and two replicates of well-watered plants (CK). A total of five seeds of each genotype were sown into plastic pots (Ø 20 × L 15 cm) filled with vermiculite and nutrient soil mixed in a 2:1 (v:v) ratio. One week after seed germination, the seedlingss were thinned to two seedlings per pot. All pots were irrigated with an equal amount of water every 3 days until the drought treatment was applied by withholding irrigation. Detailed information on the sowing, stress initiation and scoring dates is provided in Table S1. In each year each trait was scored only once, due to the large number of plant materials (95 × 2 × 3), their wide range of variation in growth speed, and strong climbing habit, which posed great challenges in scoring and plant management. The scored traits included drought-induced whole-plant wilting (Wt), the senescence of unifoliates (Scu), and stem greenness (Stg). Each of the three traits has been proven to be an excellent indicator of seedling stage drought resistance in cowpea ssp. *unguiculata* (Muchero et al., 2008; Agbicodo, 2009). The methods for scoring generally followed Muchero et al. (2008). Briefly, Wt was scored on a 0–5 scale, in which 0 referred to no sign of wilt and 5 to complete wilt. Stg was also scored on a 0–5 scale, in which 0 indicated being completely green and 5 indicated completely yellow. Scu was assessed with an index ranging from 0 to 5 that was expressed as a percentage, in which 0 corresponded to no sign of senescence and 5 to complete senescence (dead or fallen). Indices from 1 to 4 indicated that the following percentages of the leaf area were senesced: 1 ≤ 20%, 20% < 2 ≤ 40%, 40% < 3 ≤ 60%, 60% < 4 ≤ 80%. Only the stressed plants were scored because no symptom was observed in the CK group.

### Lysimetric facilities, plant growth conditions for assays, and data processing

**Figure 1:**
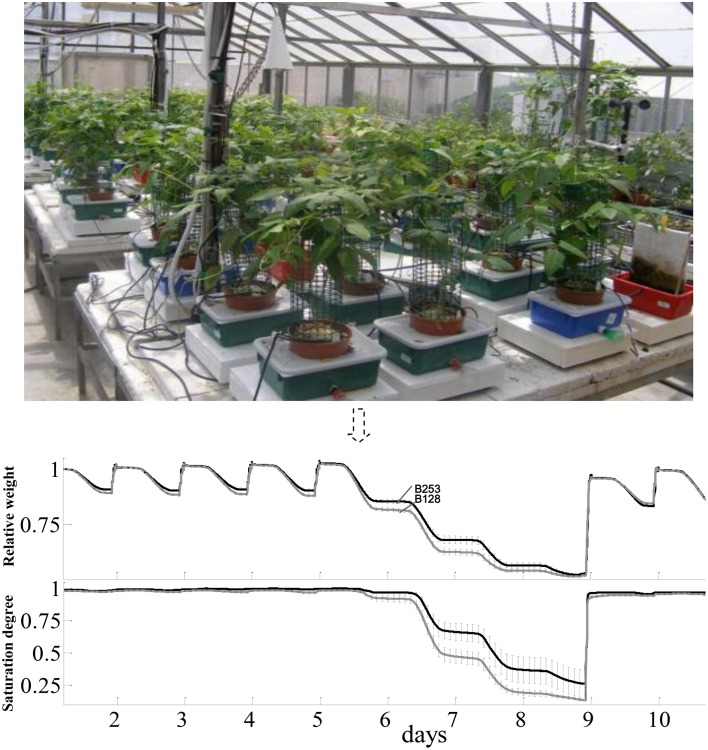
**The lysimetric system and a general description of full physiological profiling experiment**. The lysimetric system (top panel) constituted greenhouse array loaded with asparagus bean plants. This fully automated system collects data from all of the plants simultaneously. The pot-container system set on a sensitive, temperature-compensated load cell. A soil probe in each pot continuously monitors the volumetric water content and electrical conductivity of the growth media. The irrigation system is controlled by pre-programmed valves (CPV). In the bottom panel, data shown were the mean and SE of weight variation in different tested lines. Only data of B253 (black line) and B128 (gray line) were put in the figure throughout the full-irrigation and drought to make the figure clear.

Plants for lysimetric analyses were grown in a greenhouse in Rehovot, Israel (31°53′xN/34°48′E), in August, 2010 under ambient light with daily max:min temperatures of 34:15°C. The facility contains array-arranged, temperature-compensated load cells (4 L pots) with soil and environmental probes, a unique irrigation system with two dripping valves per plant to control the intensity of abiotic stress, and a central data logger and computing system (Figure 1). In details, each pot was placed in a plastic container (13 × 21.5 × 31.5 cm; height × width × length) through a hole in its top cover. A soil moisture, salinity and temperature sensor (5TE; Decagon Devices, USA) was placed in each pot. Specific coefficients of the Topp's third-order polynomial equation (Topp et al., 1980) were determined by the calibration of the sensors. The soil surface and containers were covered to avoid evaporation. The containers were filled with water daily to ensure the availability of water throughout the day and avoid the need for any supplemental irrigation that would induce weight fluctuations. A drainage hole in each container kept the water level in the container after each watering at 2 cm above the pot base. Excess irrigation was intended to prevent any accumulation of salt in the soil. This set-up ensured that the container weight decreased monotonically over the course of the day solely due to plant transpiration. After each irrigation-drainage cycle the system weight reached its original weight, but the additional weight coming from the plant biomass gain.

Five seeds were sown per cell, but only two to three uniform seedlings were retained 3 days after germination. The plants were divided into treatment and control groups, and the physiological profile of each plant was measured over an extended time period that included well-irrigated (5–7 days), treatment (10–14 days) and recovery (5–7 days) stages. Three replicates were evaluated for each line. A minimum of 7 plants from each genotype were screened simultaneously. The soil water potential, relative humidity, and temperature were recorded continuously by the probes of each individual system, and the obtained data were automatically transferred to the central computer for analysis (Campbell Scientific CR1000 Data Logger, USA). The physiological profiles of the plants were acquired based on the following whole-plant parameters: (i) daily and cumulative transpiration; (ii) daily and cumulative mass gain; and (iii) biomass WUE. The pattern of diurnal transpiration was calculated from the first derivative of the variations in smoothed weight. Plant daily transpiration (PDT) was calculated based on the difference between the load cell readings before dawn (W_*m*_) and in the evening (W_*e*_). W_*m*_ and W_*e*_ were calculated as the average weight over a 30-min period. Daily plant weight gain (Δ*P*W_*k*_) was determined based on the difference between the container weight (W_*k*+1_ - W_*k*_) on the mornings of two consecutive days, *k* + 1 and *k*, when the drainage from the container following an irrigation event had finished. Note that since drainage was controlled by a hole in the wall of the container, the observed weight difference could be attributed to plant weight gain alone. For further details concerning the sampling rate, time series analysis and noise reduction, please refer to Wallach et al. (2010).

### Trait-marker genome-wide association study (GWAS)

A trait-marker association analysis was performed using the software Tassel 2.1 (Bradbury et al., 2007) under a generalized linear model (GLM) and a mixed linear model (MLM). The parametric population structure (Q) and relative kinship matrix (K) information were according to Xu et al. (2012). Significant SNPs were defined if showing a minus log-transformed *P* > 3.

### Design and construction of a custom cDNA microarray

A custom Roche NimbleGen microarray was designed based on 29,728 unigene sequences from assembly P12 of cowpea cDNAs in the HarvEST database (Muchero et al., 2009a). The ESTs used to assemble these unigenes came from 17 cowpea cDNA libraries covering a diverse range of tissues and growth conditions. With the exception of 189 unigenes that failed to pass quality filtering for probe design, each of the unigenes was represented by a set of four 60-mer probes on the fabricated microarray. Due to high sequence similarity, 65 unigenes shared the same group of probes. Therefore, a total of 29,471 unigenes, 26,156 of which were functionally annotated, were represented on the microarray, and a total of 24 microarrays (2 genotypes × 2 tissues × 2 treatments × 3 replicates) were produced for hybridization. Ten copies of each of eight control probes derived from the yeast genome sequence with no matches to the unigene sequences were also included in the microarray.

### Plant growth conditions for microarray analyses, hybridization, and data processing

Sixteen-day-old, well-irrigated seedlings of the accessions B47 and B128 at a density of two per pot (Ø 20 cm × L 15 cm) were divided into stressed and control groups. The drought treatment was imposed by withholding irrigation. On the 15th day after treatment, two side leaflets of the first trifoliate (aerial organ) and roots (underground organ) from each stressed and control plant were harvested, and samples from the same pot were mixed for RNA extraction. The severity of drought stress was assessed by calculating the relative water content (RWC) of the middle leaflet of the first trifoliate. The formula for calculating RWC was: RWC = [(FW−DW)/(TW−DW)] × 100%, where FW, DW, and TW stand for fresh weight, dry weight, and turgid weight, respectively. Three replicates were sampled for each treatment × genotype × tissue combination. At the time of sampling, the average leaf RWC in drought-stressed B47 plants was 65.3%, which was significantly higher than that in B128 plants (52.1%; *P* < 0.01). The average leaf RWC values in the two genotypes in the control group were 83 and 81.5%, which were similar.

Total RNA was extracted using the Plant RNeasy Mini Kit (Qiagen, Hilden, Germany). RNA integrity was assessed via formaldehyde agarose gel electrophoresis, and the quantity of RNA was measured spectrophotometrically. High-quality RNA samples with an OD_260_ ≥ 2 were reverse-transcribed into cDNA using the SuperScript™ cDNA Synthesis Kit (Invitrogen, Carlsbad, CA). Microarray hybridizations were performed in the CapitalBio Corporation (Beijing, China). Briefly, labeled samples in hybridization solution were denatured at 95°C for 3 min prior to loading onto a microarray. Hybridization was performed at 42°C for 14 h with the NimbleGen Hybridization System. After two rounds of washes, the arrays were scanned using a NimbleGen MS200 scanner at a 2 μm resolution. NimbleScan software was used to extract fluorescent intensity data from the scanned images. Each scanned image was visually inspected to ensure that none, or < 1%, of the chip area had defects. Sample tracking controls (STC) were applied according to NimbleGen's instructions. The expression data from the probes were normalized using quantile normalization, and expression data for genes were generated using the Robust Multichip Average (RMA) algorithm (Irizarry et al., 2003). Multiple test corrections were performed based on the FDR (Benjamini and Hochberg, 1995). Hierarchical clustering using the average linkage method was performed with Cluster3.0 software, and the cluster results were visualized with the TreeView program (Eisen et al., 1998). Source data for each of the hybridization experiment is deposited in the NCBI Gene Expression Omnibus under the GEO series accession number GSE63636.

### Gene ontology (GO) enrichment analyses

Gene ontology (GO) enrichment analyses were performed using GOrilla (Eden et al., 2009) under a *P*-value threshold of 10E^−4^ for statistical significance. Prior to running the program, the DEGs were subjected to BLASTX searches against *Arabidopsis* protein sequences under a *P*-value cut-off of 10E^−3^ to provide legible sequence IDs for recognition in the program.

### Comparative mapping

The sequences of all DEGs and the unigenes from which SNPs associated with morphological traits were derived were subjected to BLASTX searches against the soybean genome (http://www.plantgdb.org/GmGDB/cgi-bin/blastGDB.pl) to identify the LD relationships of their orthologs. The criteria for declaring orthologous sequences were as follows: length of aligned sequence ≥80 bp, and an *e*-value ≤ *e*^−10^. Wherever multiple hits occurred, only the best hit was considered.

## Results

### Natural variation in drought responses and the marker-trait associations

**Figure 2:**
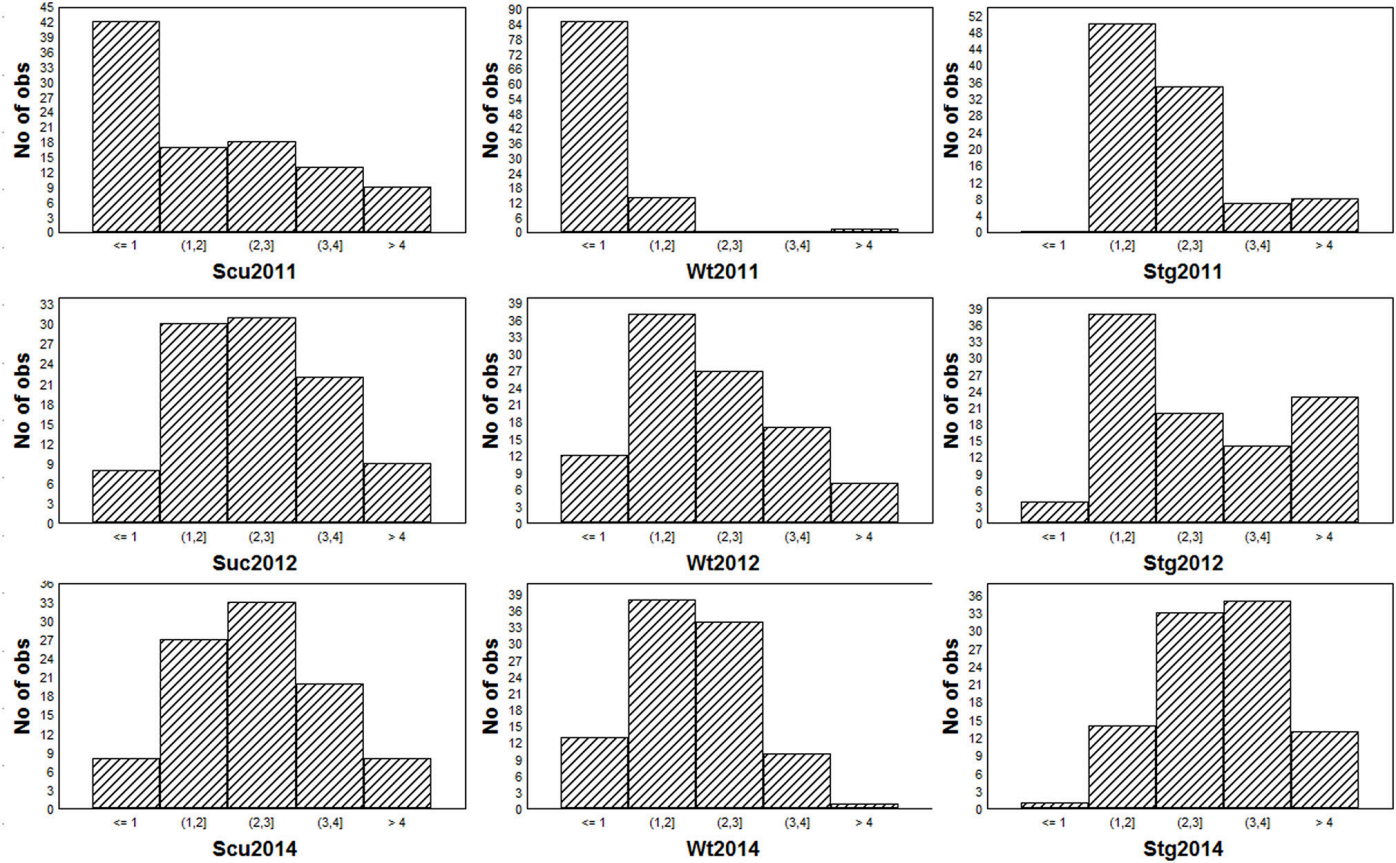
**Frequency distribution of the three morphological traits in the three trials**.

To obtain global insight into the natural variation in drought resistances, three morphological traits, viz. drought-induced whole-plant wilting (Wt), the senescence of unifoliates (Scu), and stem greenness (Stg) were scored across the 95-accession Chinese asparagus bean mini-core diversity panel in the years 2011, 2012, and 2014. Each year, all three traits exhibited a range of variation from grades 0 (the lowest) to 5 (the highest; Figure 2), demonstrating a broad genotypic variability of drought resistance in the germplasm. Stg showed the highest correlation coefficients over the course of the experiment (Table 1), which is in agreement with Muchero et al. (2008). By year, the pairwise correlation coefficients between the three traits were highest in 2012 (0.449–0.724), followed by 2014 (0.319–8–0.657), while they were lowest in 2011 (0.251–0.312). A higher level of skewness of the data was observed for the 2011 data, which could be attributed to scoring the traits in an earlier stage of a different season compared with later years.

**Table 1:** **Significant correlation co-efficiencies of the traits visually scored in different years (*P* ≤ 0.05)**.

	**Scu2011**	**Wt2011**	**Stg2011**	**Scu2012**	**Wt2012**	**Stg2012**	**Scu2014**	**Wt2014**
Scu2011^a^								
Wt2011	0.251							
Stg2011	–^b^	0.312						
Scu2012	0.277	0.256	0.376					
Wt2012	-	0.318	0.444	0.510				
Stg2012	-	0.327	0.581	0.449	0.724			
Scu2014	0.232	–	–	0.361	0.256			
Wt2014	–	0.228	–	0.271	0.275	0.338	0.578	
Stg2014	–	0.242	0.393	0.339	0.407	0.558	0.318	0.657

a *Scu, senescence of unifoliates; Wt, whole-plant wilting; Stg, stem greenness*.

b *No significant correlation detected*.

**Figure 3:**
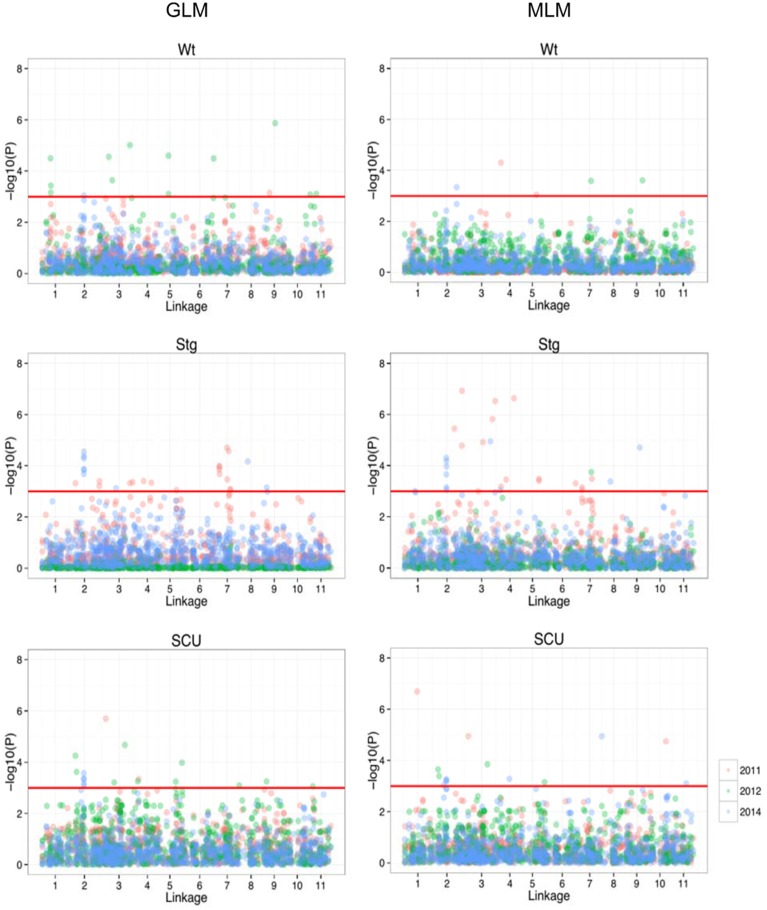
**Manhattan plot of the genome-wide associations in drought resistance traits**. On the left are the results from GLM, and on the right are results from MLM. In each panel, the minus log-transformed *P*-value for each SNP for association with drought resistance in each year was presented (as indicated in different colors).

Using previous genome-wide SNPs data for the population (Xu et al., 2012), a GWAS was performed under both a generalized-linear model (GLM) and mixed-linear model (MLM) to search for SNP loci associated with the three traits. Under the given statistical threshold, 4, 26, and 11 SNP loci were found to be consistently associated with Wt, Scu, and Stg (*P* < 0.01), respectively (Figure 3, Table 2). Two SNPs were associated with more than one trait. These SNPs were distributed among 10 of the 11 linkage groups (LGs) in the cowpea consensus genetic map, with the exception of LG1 (Muchero et al., 2009b), and each explained a moderate to small amount of the observed phenotypic variation. The SNP loci in LG2, 3, 4, 5, and 8 formed several clusters in which neighboring loci were < 5 cm apart. Ten significant SNPs were found to be coincident with SNPs for adult plant drought resistance detected in the African cowpea germplasm (Muchero et al., 2013). Eleven SNPs fell within the regions of drought resistance QTLs previously identified in African cowpea (Muchero et al., 2009a).

**Table 2:** **Marker-trait associations detected by GWAS**.

**SNP loci**	**Traits associated^a^**	**Experiment and methods detected^b^**	**Linkage group**	**Position (cm)**	**Associated drought resistance traits in African cowpea**	**Fall into IT93K-503-1 X CB46 QTLs**
1_1258	Stg	G2011, M2011, 2012	LG2	16.45		
1_1181	SCU	G2012, 2014, M2014	LG2	35.49		
1_0693	SCU	G2011, 2014, M2014	LG2	35.76		
1_1140	SCU	G2012, 2014, M2014	LG2	35.76		
1_1352	SCU	M2011, 2014	LG2	57.49	Senescence	*Dro-9*
1_1021	SCU	G2011, 2014	LG2	71.25		*Dro-9*
1_1021	Stg	G2011, 2014, M2011	LG2	71.25		
1_1481	Stg	G2011, M2011, 2014	LG2	71.66		
1_0724	SCU	G2011, 2012, M2011	LG3	11.96		
1_1483	Stg	G2011, 2014	LG3	33.34		
1_0636	SCU	G2011, 2012	LG3	38.59	Biomass	
1_1349	SCU	G2011, 2012	LG3	39.83	Senescence	
1_0758	SCU	G2011, 2012	LG3	44.93		
1_1022	SCU	G2011, 2012	LG3	44.93		
1_1427	SCU	G2011, 2012	LG3	44.93		
1_0086	SCU	G2011, 2012	LG3	45.02		
1_0064	SCU	G2011, 2012	LG3	45.73		
1_1005	Wt	G2011, 2014	LG3	52.26		
1_0380	Stg	G2011, M2011, 2014	LG3	73.42		
1_1162	Stg	G2011, M2011	LG3	73.79		
1_0888	SCU	G2011, 2012, 2014, M 2012, 2014	LG4	0.72	Seed weight per plant	
1_1147	SCU	G2011, 2012, 2014, M 2011	LG4	2.77	Grain yield	
1_0128	Stg	G2011, 2014	LG4	29.51		
1_0205	SCU	G2011, 2012, M2012	LG5	43.29		
1_0127	Stg	G2011, 2014	LG5	44.42	Seed weight per plant	
1_0032	SCU	G2011, 2012, M2012	LG5	45.27		
1_0664	SCU	G2011, 2012	LG6	59.00		
1_1057	Wt	G2011, M2012	LG7	27.33	100-seed weight	*Dro1*
1_1057	Stg	G2011, M2011, 2012	LG7	27.33		*Dro1*
1_0298	SCU	G2012, M2012, 2014	LG8	3.80		
1_0771	SCU	G2011, 2012	LG8	10.64		
1_0838	Wt	G2011, 2014	LG8	42.11	Seed number per plant	*Dro-3*
1_1374	SCU	G2011, 2012	LG8	45.26		*Dro-3*
1_1167	SCU	M2011, 2014	LG9	0.20		
1_0097	SCU	M2011, 2014	LG9	8.60	Grain yield	
1_0474	SCU	M2011, 2014	LG9	40.27		
1_0759	Stg	G2011, M2011, 2014	LG10	40.74	Seed number per pod	*Dro-3*
1_0603	SCU	G2011, 2012, M2012	LG11	4.60		*Dro-3*
1_0905	SCU	G2011, 2012	LG11	5.71		*Dro-3*
1_0486	Wt	G2012, M2014	LG11	23.14		*Dro-3*
1_0274	Stg	G2012, M2014	LG11	26.78		*Dro-3*

a *Scu, senescence of unifoliates; Wt, whole-plant wilting; Stg, stem greenness*.

b *G, GLM; M, MLM*.

### Whole-plant water relations in four independent accessions under well-watered and drought conditions

**Figure 4:**
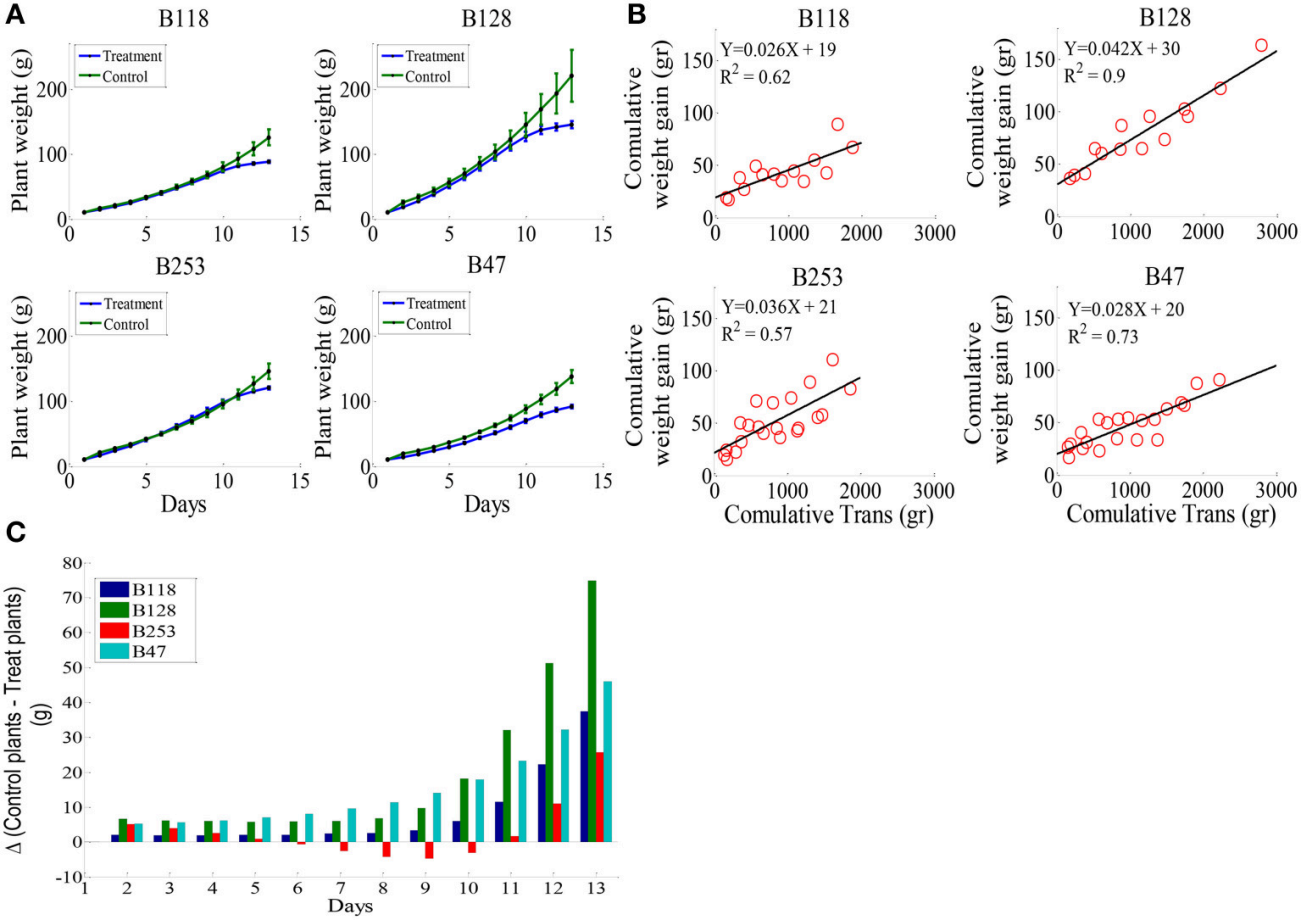
**Whole-plant growth rate and water use efficiency (WUE) in the four lines**. **(A)**, Mean and SE of the whole-plant cumulative biomass. **(B)**, Plant WUE, defined as the ratio between plant weight gain (Δ_*PW*_) and the amount of water transpired. The WUE of each line was determined by fitting a linear curve for the cumulative plant weight gain during the pretreatment stage vs. cumulative water transpiration. **(C)**, The loss of weight gain due to drought treatment (bigger bars indicate greater loss). A minimum of seven plants from each genotype were screened simultaneously.

**Figure 5:**
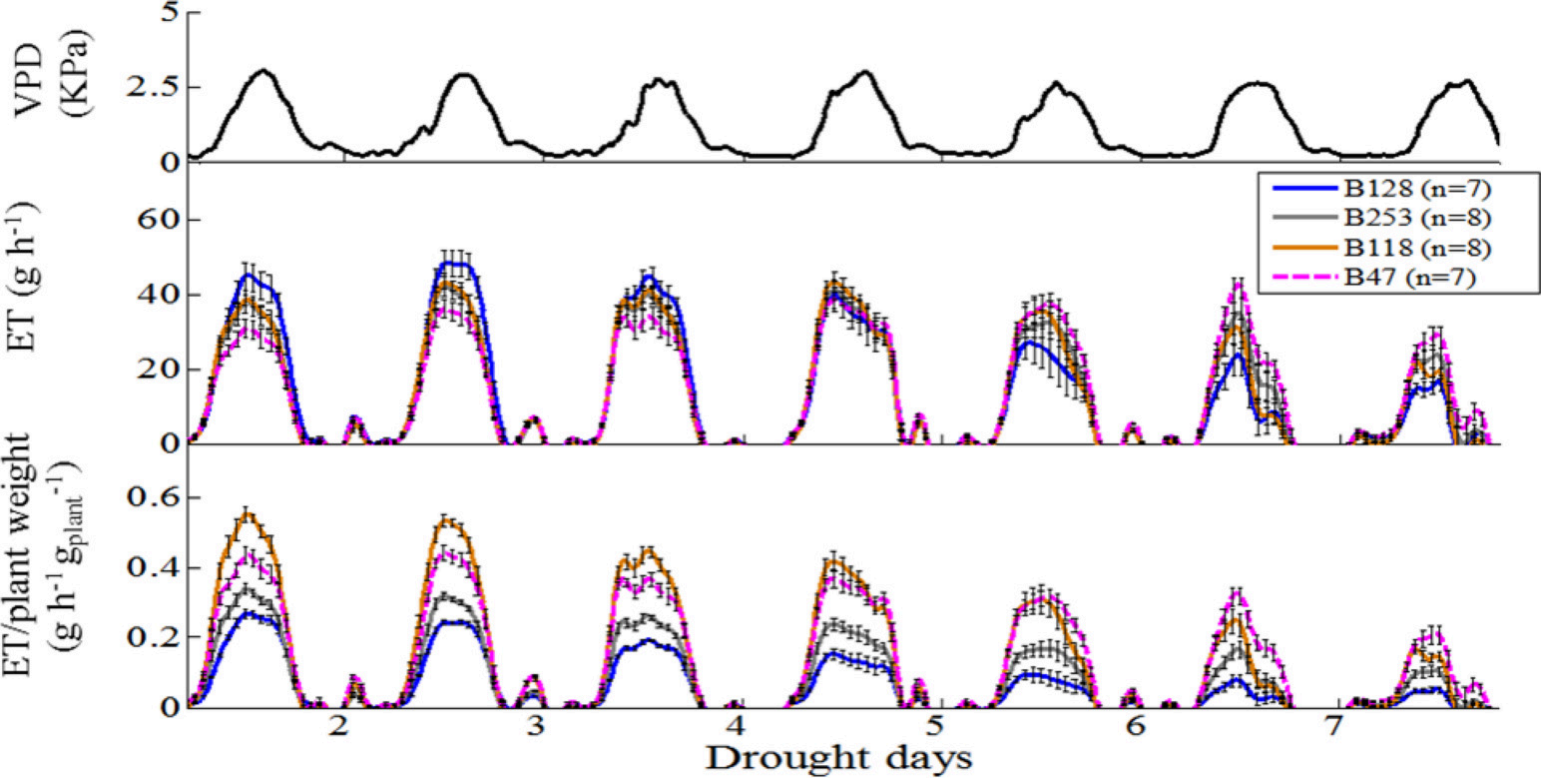
**Whole-plant response to ambient (atmosphere and soil) variation, normalized to plant weight and the vapor pressure deficit (VPD)**. **Top panel**, Daily VPD during seven consecutive days of drought. **Middle panel**, Mean and SE of the rate of diurnal water loss (transpiration) in whole plants during 7 days of drought treatment, calculated as the first derivative of the measured weight loss. **Bottom panel**, Mean and SE of the rate of whole-plant transpiration (in the same plants as above), normalized to the plant weight. A minimum of seven plants from each genotype were screened simultaneously.

According to the different performances in visual phenotyping (Table 3) and the representativeness of the Chinese asparagus bean germplasm, four accessions, viz. B47, B118, B128, and B253, were employed or whole-plant water relation analysis using a lysimetric system (Figure 1). All four genotypes exhibited a diurnal rhythm of weight changes under WW conditions, with weight loss occurring only during daytime (Figure 1). This observation suggests that the stomata of all genotypes were relatively closed at night. The plant growth rate and water use efficiency (WUE) exhibited major differences between lines (Figures 4A,B). B128 presented the fastest growth rate and the highest WUE under well-watered conditions; however, drought treatment caused the greatest loss of growth potential in this line (Figure 4C). B47 showed a slow growth rate and moderate WUE with a less drastic loss of growth potential in response to drought treatment. B118 and B253 presented greater stability of these parameters. Observations of whole-plant transpiration (ET) and ET normalized to plant size (ET/weight; ETW) across depletion of the soil water content (SWC) revealed that plant transpiration was size dependent (Figure 5). B128, which showed the highest ET under WW conditions, presented the lowest water loss per unit of mass (ETW), while the opposite pattern was observed for B118 and B47.

**Table 3:** **Scores of Scu, Stg and Wt in B47, B118, B128, and B253 under drought stresses**.

**Genotype**	**Wt^a^**			**Stg^a^**			**Scu^a^**		
	**2011**	**2012**	**2014**	**2011**	**2012**	**2014**	**2011**	**2012**	**2014**
B47	0.33	1	0.4	2	1.67	2	1	2	1.6
B118	0.33	1.67	2.67	2	2.67	3.33	3	5	3.33
B128	0.33	1.5	3	2.33	3	3.33	1	2.33	2.8
B253	0.67	3.67	1.67	4.66	5	5	1	3.67	2.33

a *Scu, senescence of unifoliates; Wt, whole-plant wilting; Stg, stem greenness. Data reported for each year are the means of three replicates*.

**Figure 6:**
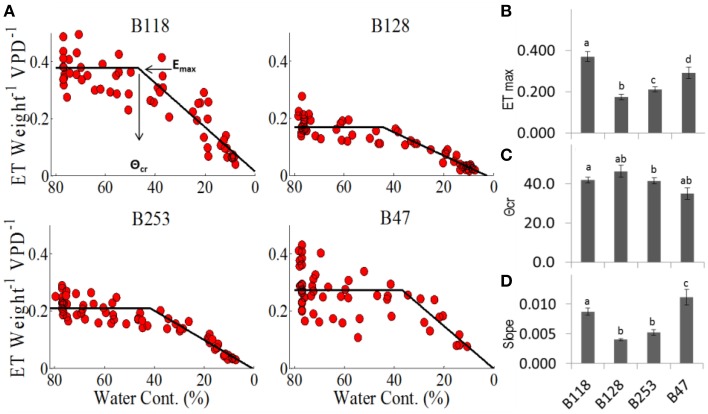
**Midday transpiration vs. soil water contents. (A)** Midday whole-plant transpiration (normalized to weight and VPD) as a function of the relative soil water content. The specific values for each cultivar are as follows: **(B)** E_*max*_ is the maximum transpiration before it reaches **(C)** the critical soil water content, θ_cr_, after which E decreases, as described by the slope **(D)**. Different letters represent significant differences (*t*-test, *P* < 0.05). A minimum of seven plants from each genotype were screened simultaneously.

For each genotype, the plot of TW vs. SWC revealed a distinct, constant midday transpiration level (*E*_max_) until the soil moisture content reached a critical value (θ_cr_), when a decrease in the transpiration rate took place (Figures 6A,B). This ETW (SWC) pattern indicates that soil water availability becomes a limiting factor only when SWC < θ_cr_, and each accession has a unique capacity to maintain the maximum transpiration rate under progressive soil water depletion. The lines B47 and B128 appeared to be the most and least sensitive to soil moisture deficits, as they exhibited the highest and lowest θ_cr_ values (Figures 6C,D). B47 also showed the highest rate of ET decline after θ_cr_ was reached (Figure 6D), suggesting that this line achieved the most effective dehydration avoidance response.

### Comparison of drought-responsive transcriptomes between B47 and B128

**Figure 7:**
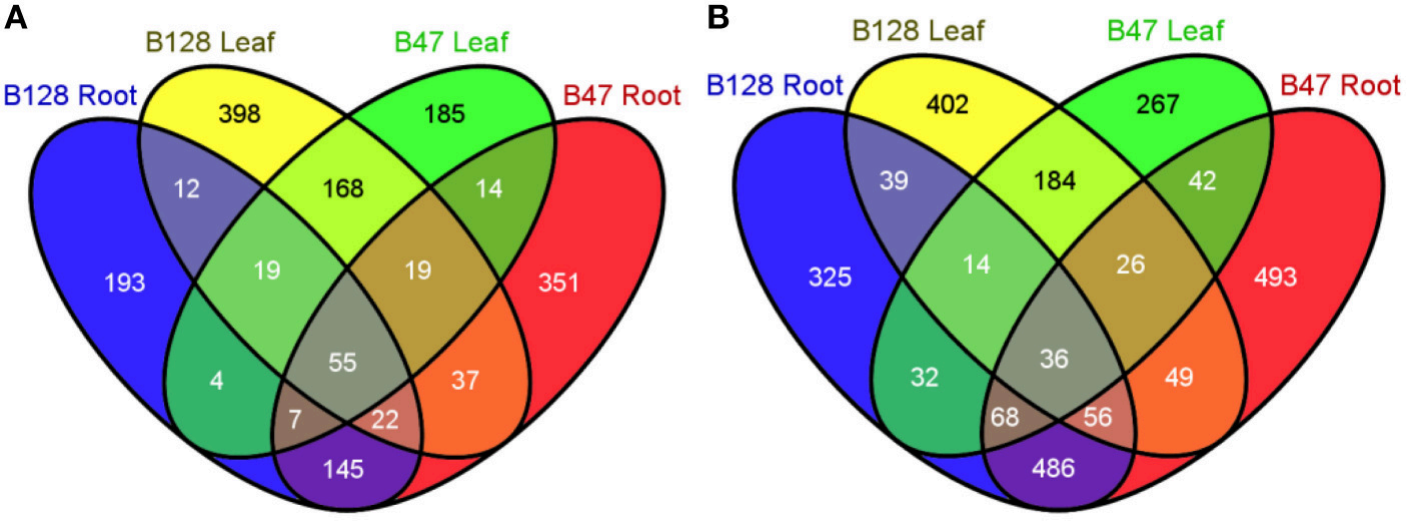
**Venn diagrams showing the number and relationships of drought-regulated genes in B47 and B128**. **(A)**, up-regulated genes under drought stress; **(B)**, down-regulated genes under drought stress.

Because B47 and B128 displayed major differences in the regulation of the whole-plant water status under soil water deficits, the drought-responsive transcriptomes of their leaf and root organs were compared using a custom microarray hybridization method. The consistency of the microarray data was confirmed by high pair-wise correlation coefficients (*r*, 0.944–0.993) between replicates (Table S2). The number, identity and relationships of up- and down-regulated (fold change ≥2, FDR ≤ 0.01) genes in B47 and B128 are shown in Venn diagrams (Figure 7) as well as Tables S3, S4. Gene ontology (GO) enrichment analysis of the differentially expressed genes (DEGs) clearly demonstrated a much more complicated GO enrichment network among down-regulated genes as compared with up-regulated genes, particularly in roots (Figure S1, Table S5), which appeared characteristic to asparagus bean. Some well-known GO terms for plant drought responses, such as the “response to water deprivation,” “response to lipid,” and “response to abscisic acid,” were enriched in both genotypes. Known protective or regulatory genes including *ABI1, ABF2, PP2CA*, and *NCED5* were annotated by these GO terms. These results revealed that some common drought resistance pathways such as the ABA and phosphate lipid signaling are also fundamental in asparagus bean.

Some GO terms were enriched in a genotype-specific manner. Among the genes up-regulated in leaves of B47, GO terms were enriched mostly for the responses to water deprivation, ABA and oxygen compounds, whereas in B128 they comprised more items like the “hyperosmotic salinity response,” “response to inorganic substance,” and “response to alcohol.” Among the down-regulated genes in the same organ, the GO terms enriched in B128 were related to photosynthesis, ion transportation and cellular homeostasis, whereas in B47 no significant enrichment of photosynthesis genes was noted. Rather, additional GO terms such as the “hormone-mediated signaling pathway,” “defense response,” and “response to jasmonic acid” were found to be specifically enriched in B47. Given the differential regulation of whole-plant water relations between the two line expressed as, for instance, the different leaf water contents and yellowish/wilting phenotypes, the GO enrichment patterns were in good accordance with each line's physiological characteristics. More interestingly, GO terms related to ethylene synthesis/responses were enriched in both B47 and B128, but these effects were observed in different organs (the leaves of B47 and roots of B128). Karrikin, a newly discovered plant growth regulator and smoke-derived abiotic signal (Chiwocha et al., 2009), might also be involved in the genotypic difference of drought resistances, as the GO term “response to karrikin” was specifically enriched in leaves of B128.

**Figure 8:**
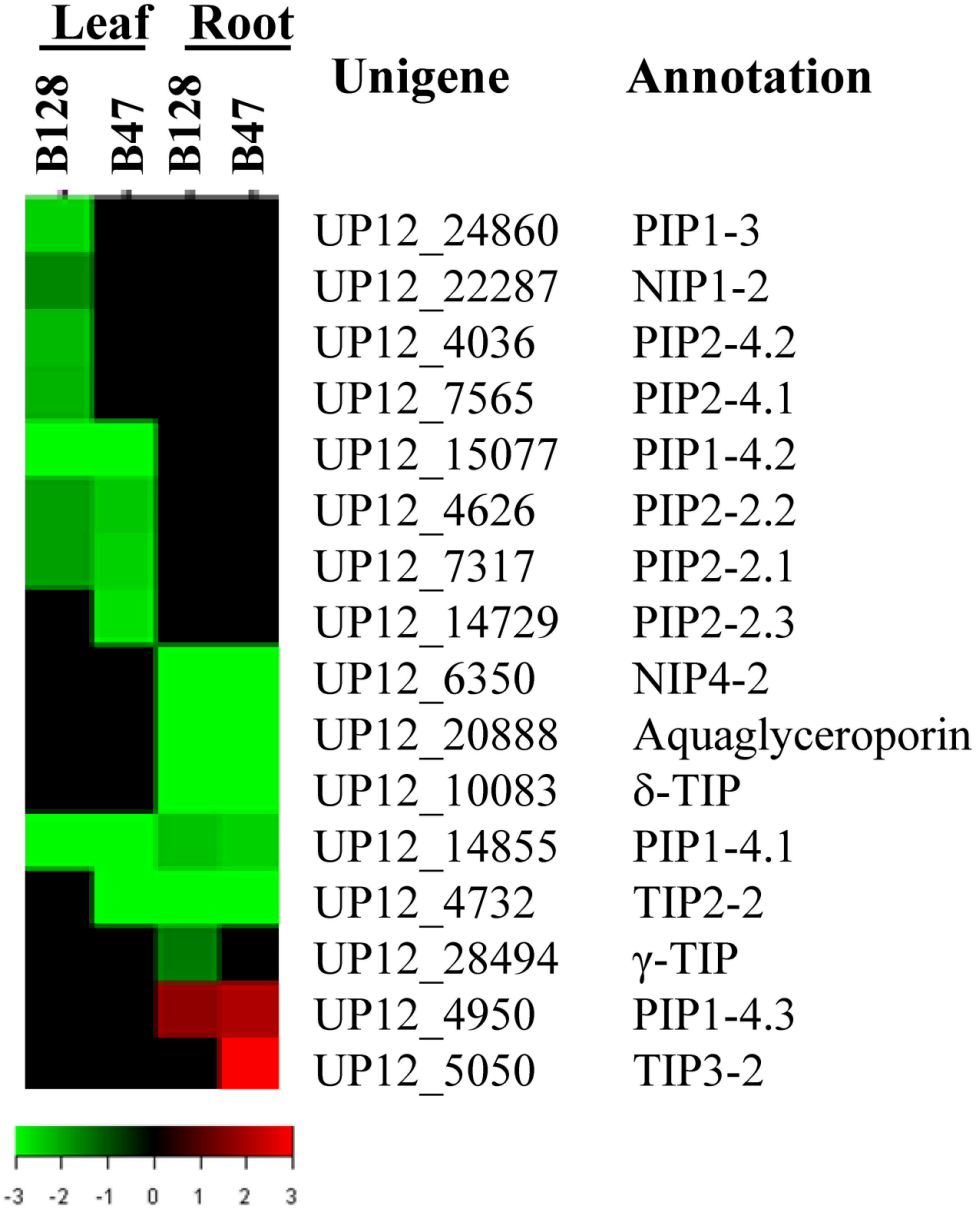
**TreeView presentation of the expression profiles of 16 drought-responsive aquaporin genes**. The genes that showed a ≥ 2-fold change in transcript abundance and an FDR < 0.01 are included.

Because recent investigations have pointed to a possible key role of aquaporins (AQPs, water channels that are critical to cross-membrane water exchange) in determining the genotypic variation of whole-plant water relations (Peng et al., 2007; Sade et al., 2010), we specifically assessed the expression of cowpea aquaporin genes in response to drought stress. There were 52 putative AQP genes represented on the microarray. It was shown that the transcript abundance of 16AQPs was regulated by drought in at least one genotype × tissue × treatment combination, with the majority being down regulated (Figure 8). Eight and six AQP genes were regulated in a leaf- or root-specific manner, respectively. Seven AQP genes exhibited a genotypic-dependent response to drought stress (five in B128 and two in B47). The latter included TIP3-2, one of the only two AQP genes found to be up regulated in this experiment.

### Differentially expressed genes (DEGs) that show also genetic associations with drought resistance traits

DEGs that fall into a QTL interval or linkage disequilibrium (LD) block for a certain trait are logically considered valuable candidate genes for that trait (Muchero et al., 2010; Stanton-Geddes et al., 2013). Here the same strategy was applied to narrow down the list of DEGs that are more likely to be causal factors for the genotypic differences in drought responses. Firstly, the DEGs with known locations in the cowpea consensus genetic map (Muchero et al., 2009b) were analyzed for co-localization with the significant SNPs. Given the average LD decay distance of approximately 2 cm across the asparagus bean genome (Xu et al., 2012), DEGs that were coincident with or < 2 cm from significant SNPs were considered useful candidates. According to this standard, 31 DEGs were detected (Figure S2, Table S6). The proteins encoded by these DEGs include known abiotic stress-related proteins, such as alcohol dehydrogenase, Ca^2+^-binding protein 1, non-specific lipid-transfer proteins, chalcone isomerase 2, as well as various types of transcription factors.

Because the majority of the DEGs have not been positioned in the cowpea consensus map thus could not be detected by the above method, a comparative genomic approach using the soybean genome sequence was employed to determine the LD relationships of the DEGs and significant SNPs. Here, a maximum physical distance of 1.8 Mb, which corresponds to approximately 2 cm in genetic distance in the cowpea genome, was allowed to define the LD status of a DEG and a SNP. As a result, 276 DEGs, including four drought-regulated AQP genes (drAQPs), viz. *PIP1-4.2, PIP2-2.2*, γ*-TIP*, and *aquaglyceroporin*, were detected (Table S7, Figure S2). Other highly interesting DEGs include those encoding the abscisic acid receptor PYL5, dehydration-responsive element-binding protein 3, a senescence/dehydration related protein, and more. Taken together, these results provide useful information on candidate genes for the causal genes underlying the genotypic differences in drought resistance.

## Discussion

### The ssp. *sesquipedalis* subgene pool has maintained high natural variation in soil drought responses despite severe domestication bottleneck

Asparagus bean (the ssp. *sesquipedialis*) was derived from domesticated ssp. *unguicualta* that represents only a small portion of the genetic variation present in African cowpea. Hence, it is thought to have gone through a dual domestication that lowered its genetic diversity profoundly (Fang et al., 2007; Xu et al., 2012). Through the large-scale investigation of whole-plant morphological traits over the course of the experiment, we obtained evidence showing that high natural variation has been maintained in the Chinese asparagus bean germplasm despite severe domestication bottleneck. This finding should be partly explained by the fact that the artificial selection of asparagus bean plants in the more humid Asia (compared to west Africa) has primarily targeted pod quality traits (e.g., length, softness and flavor) and with less attention paid to drought resistance. In further support of this explanation, it has been found that drought-resistant traits and pod traits are genetically governed by largely independent regions/loci (Hu, 2011; Xu et al., 2013; this study). This genetic architecture also indicates that the prospects for developing more drought-resistant varieties without impairing pod quality are promising. However, these results should still be interpreted with caution due to the lack of repeats over time. The coincidence of many SNP loci detected for seedling stage drought-resistance (this study) and post-flowering stage resistance (Muchero et al., 2009a, 2013) demonstrates the feasibility of selecting for adult drought resistance at the seedling stage with the assistance of SNP markers.

### High and divergent sensitivity to soil water content provides an important mechanism configuring genotypic specific drought responses

Like the better-characterized drought-resistant crop species chickpea (Zaman-Allah et al., 2011) and pearl millet (Kholová et al., 2010), dehydration avoidance has been suggested to be the primary mechanism for achieving drought resistance in African cowpea (Bates and Hall, 1982; Likoswe and Lawn, 2008). Here we further revealed that a relatively high θ_cr_ value held true for all four genotypes investigated, indicating that asparagus bean is inherently sensitive to soil water deficits. This characteristic is supposed to confer asparagus bean plants effective drought avoidance when facing soil drought. On the other hand, genotypic differences in transpiration patterns and the critical soil moisture threshold are apparent among varieties. Our results demonstrated that B47 exhibited relatively low growth and transpiration rates and the highest sensitivity to changes in soil water contents, with its stomatal aperture decreasing with the fastest slope after reaching its critical point. This response would allow more efficient water saving during drought spells and would be accompanied by a decrease in carbon fixation (Aharon et al., 2003). Morphologically, B47 stayed stem-green much longer and had less senesced unifoliates. Based on these observations, B47 fits well the feature of ‘type I’ drought resistance. On the contrary, B128 showed a more yellowish stem and rapid senescence of unifoliates. Physiologically, it presented less conservative behavior with a faster growth rate and higher transpiration, suggesting that this line may exhibit favorable characteristics under high to moderate water conditions (not below its θ_cr_), but not in severe drought environments (Moshelion et al., 2015). Hence, B128 could represent a “type II” drought resistant line. Clearly, the historical classification of the two types of drought resistance in cowpea may in essence refers to the two various types of drought responses in relation to soil drought sensitivity, with a type I resistance focusing more on the physiological avoidance to soil drought and a type II resistance more on the agricultural outcomes on a final yield basis. Here it is also noteworthy that the methodology of plotting ETW vs. SWC presented more physiological relevance for comparisons of transpiration regulation behavior among the various accessions than plotting of ETW over time, which can be explained by the fact that plants that transpire more also reduce the soil water content faster and are therefore subjected to water shortages sooner.

Understanding the different concepts of physiological and agronomical drought resistances is important to agricultural practices. Frequently, to improve the drought resistance of a crop, breeders tend to perform crosses with physiologically highly resistant lines, i.e., those showing swift and profound responses to drought. However, this practice often leads to undesired results because plant resistance against soil drought in the field is tightly associated with overall plant growth habits, and hence, every claim of resistance enhancement needs to be tested on a crop yield basis. In the case of asparagus bean in Asia, because only mild and intermittent soil drought may occur during the asparagus bean growing season, genotypes such as the physiologically highly resistant line B47 (type I) may in fact not be advantageous agriculturally, as this resistance tends to inhibit growth more than necessary, leading to greater yield losses under non-lethal stresses. On the contrary, introgression of type II drought responsive traits such as more stable transpiration would be more desirable in this particular eco-region (McDowell et al., 2008). Noteworthy, it is a prerequisite to determine the critical soil moisture threshold at which type II genotypes lose their agronomic advantage, as it is the basis for selecting suitable donor accessions under a specific water shortage regime.

### Candidate genes underlying the genotypic differences in whole-plant water relations in response to soil drought

An important aim of this study was to delimit genes underlying the genotypic differences in whole-plant water relations under drought conditions. By narrowing down the list of DEGs based on the GWAS result, 296 unique DEGs were identified to be in LD with the 39 significant SNPs. These DEGs cover diverse functional categories, including hormone responses, water and nutrition transportations, cell wall adjustment, transcriptional factors and more, which give valuable directions for the elucidation of the causal factors causing the genotypic differences in drought responses. The genotype-specific enrichment of the “cell wall disassembly” and “cell wall modification involved in abscission” among up-regulated genes suggests a role of the cell wall adjustment in providing different levels of plasticity for the resistance to water deficit (Neumann, 1995). The association of B47-specificallyregulated SAUR-like gene with drought resistance indicates the involvement of auxin signals in the genotypic differences of drought responses. More interestingly, the differential tissue-specific enrichment of the ethylene biosynthesis/response GO terms among the down-regulated genes is in support of the recent finding that reduced level of ethylene lead to a more resistant phenotype to drought (Skirycz et al., 2011), and might be indicative of the differential root-to-shoot signaling of hydraulic status used by the two lines (Schachtman and Goodger, 2008).

AQPs constitute a highly divergent protein family (Chaumont et al., 2001) and work at multiple levels to regulate plant hydraulic relations. The general trend of down-regulation of asparagus bean AQP genes by drought stresses is in agreement with previous observations made in Arabidopsis and rice (Guo et al., 2006; Alexandersson et al., 2010). The identification of four drought-regulated AQP genes (*PIP1-4.2, PIP2-2.2*, γ*-TIP*, and *aquaglyceroporin*) that show genetic association with drought-resistance traits directs to a more focused scope for future validation of the functional involvement of AQPs in the natural variation of drought responses. Specifically, the asparagus bean *PIP1-4.2* gene was found to be depressed only in the less drought-avoidant line B128. This behavior appears similar to that of the rice *OsPIP1-3* gene, which was induced only in a more drought-avoidant upland cultivar (Lian et al., 2006), and may indicate a similar role of the two genes. Additional future work will be required to explicitly reveal the roles of certain AQP genes in adjusting the plant hydraulic framework under drought conditions.

It must be acknowledged that current strategies to identify candidate genes would have missed some real genes responsible for the genotypic difference, which may have functional polymorphisms but no differences in expression. The still incomplete genome coverage of the GWAS platform currently available for asparagus bean would have also left some genome regions contributing to the phenotypic variation of drought responses undetected. A next generation of genomic resources for cowpea/asparagus bean to come (Close et al., 2015) will lead to a more thorough understanding of the basis of the genotypic difference in drought resistance in this species.

## Author contributions

All authors contributed to the experiment and the manuscript. PX, MM, and GL designed the experiment and drafted the manuscript. PX and OH conducted the experimental work. PX, XW, BW, XW, and ZL carried out the field work. PX, OH, JL, MM, and RW analyzed the data. All authors have read and approved the final manuscript.

### Conflict of interest statement

The authors declare that the research was conducted in the absence of any commercial or financial relationships that could be construed as a potential conflict of interest.

## Supplementary material

Figure S1 **Diagram of GO term enrichments. (A)**, GO term enrichment represented by up-regulated genes in leaves of the two lines; **(B)**, GO term enrichment represented by down-regulated genes in leaves of the two lines; **(C)**, GO term enrichment represented by up-regulated genes in roots of the two lines; **(D)**, GO term enrichment represented by down-regulated genes in roots of the two lines. Enrichment was generated using GOrilla with a *P* ≤ 10^−4^.

Figure S2 **Differentially expressed genes (DEGs) that are in linkage disequilibrium (LD) with the SNPs associated with drought resistance**. On the left is the SNP-based genetic linkage map. On the right is the soybean chromosome regions corresponding to the cowpea linkage groups (note that for clarity only orthologous of drAQPs were shown). The loci marked with blue triangles are SNPs significantly associated with drought resistance (*P* < 0.01). The loci marked with arrows represent DEGs that are in LD with the significant SNPs. Different colors and directions of the arrows indicate the various patterns of transcriptional regulation of the DEGs. Upward arrows: up-regulated DEGs. Downward arrows: up-regulated DEGs. Red arrows: regulated in B47 only. Red arrows: regulated in B128 only. Dark arrows: regulated in both genotypes.

Table S1 **Dates of seed sowing and phenotyping in the course of the experiment**.

Table S2 **Pairwise correlation coefficients for the microarray data among each of the three replicates**. See below for descriptions of xp1-24.

Table S3 **Genes that are drought-regulated in both B47 and B128**. In red are up-regulated genes and in green down-regulated genes. FC, fold change.

Table S4 **Genes that show genotypic-specific regulation by drought**. In red are up-regulated genes and in green down-regulated genes. FC, fold change.

Table S5 **List of the GO terms enriched for drought-responsive genes in different tissues of B47 and B128**. Lf, leaf; Rt, root.

Table S6 **Mapped DEGs that are in LD with the significant SNPs detected by GWAS**.

Table S7 **DEGs that are in LD with the significant SNPs detected by GWAS via a comparative genomic approach**.

## Abbreviations

AQPs: aquaporins
DEG: differentially expressed genes
ET: whole-plant transpiration
GLM: generalized-linear model
GO: Gene ontology
GWAS: genome-wide association study
LD: linkage disequilibrium
LGs: linkage groups
MLM: mixed-linear model
RWC: relative water content
Scu: the senescence of unifoliates
Stg: stem greenness
SWC: soil water content
Wt: whole-plant wilting
WUE: water use efficiency.
