# Supplementary material for: Natural variation and gene regulatory basis for the responses of asparagus beans to soil drought
Source: Front Plant Sci. 2015 Oct 27;6:891. doi: 10.3389/fpls.2015.00891 (PMC4621818; doi:10.3389/fpls.2015.00891)
Supplement: Figure S1 — Diagram of GO term enrichments. (A), GO term enrichment represented by up-regulated genes in leaves of the two lines; (B), GO term enrichment represented by down-regulated genes in leaves of the two lines; (C), GO term enrichment represented by up-regulated genes in roots of the two lines; (D), GO term enrichment represented by down-regulated genes in roots of the two lines. Enrichment was generated using GOrilla with a P ≤ 10−4. [file Image1.PDF]

A

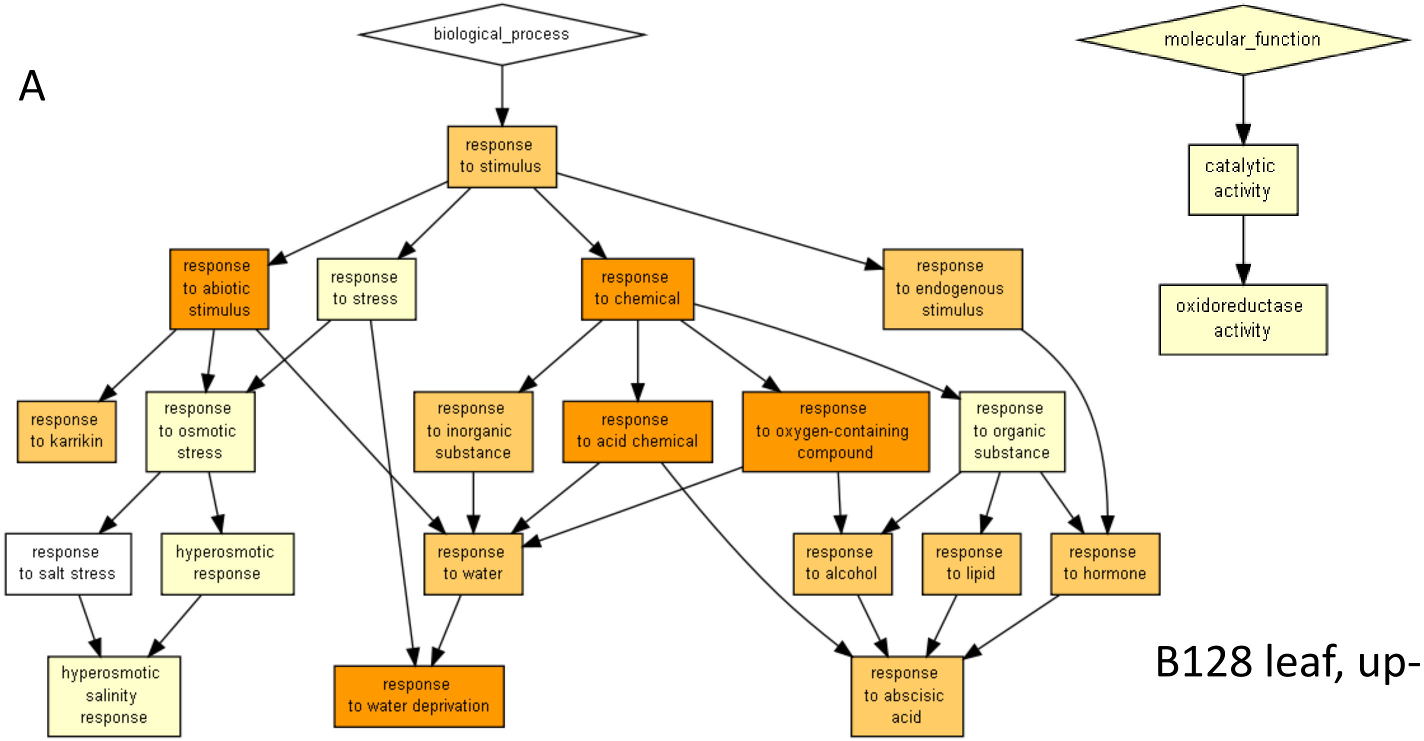

B128 leaf, up-regulated genes

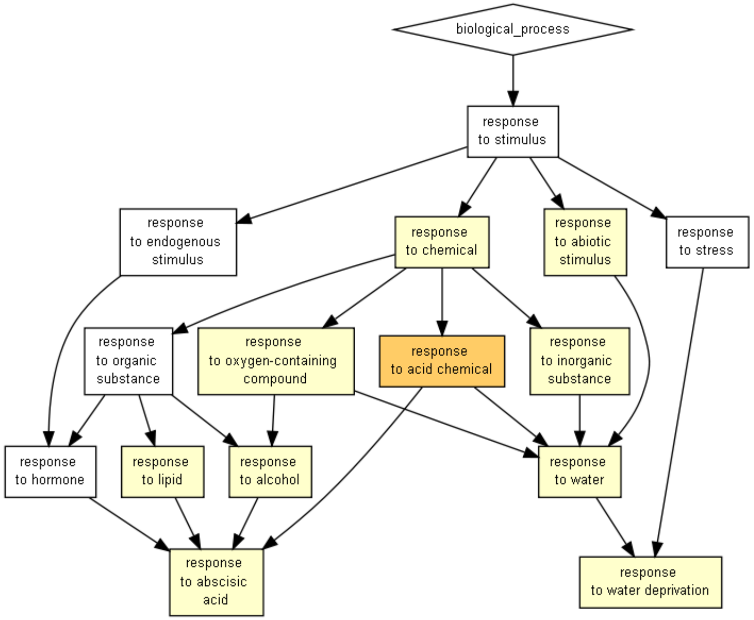

B47 leaf , up-regulated genes

B

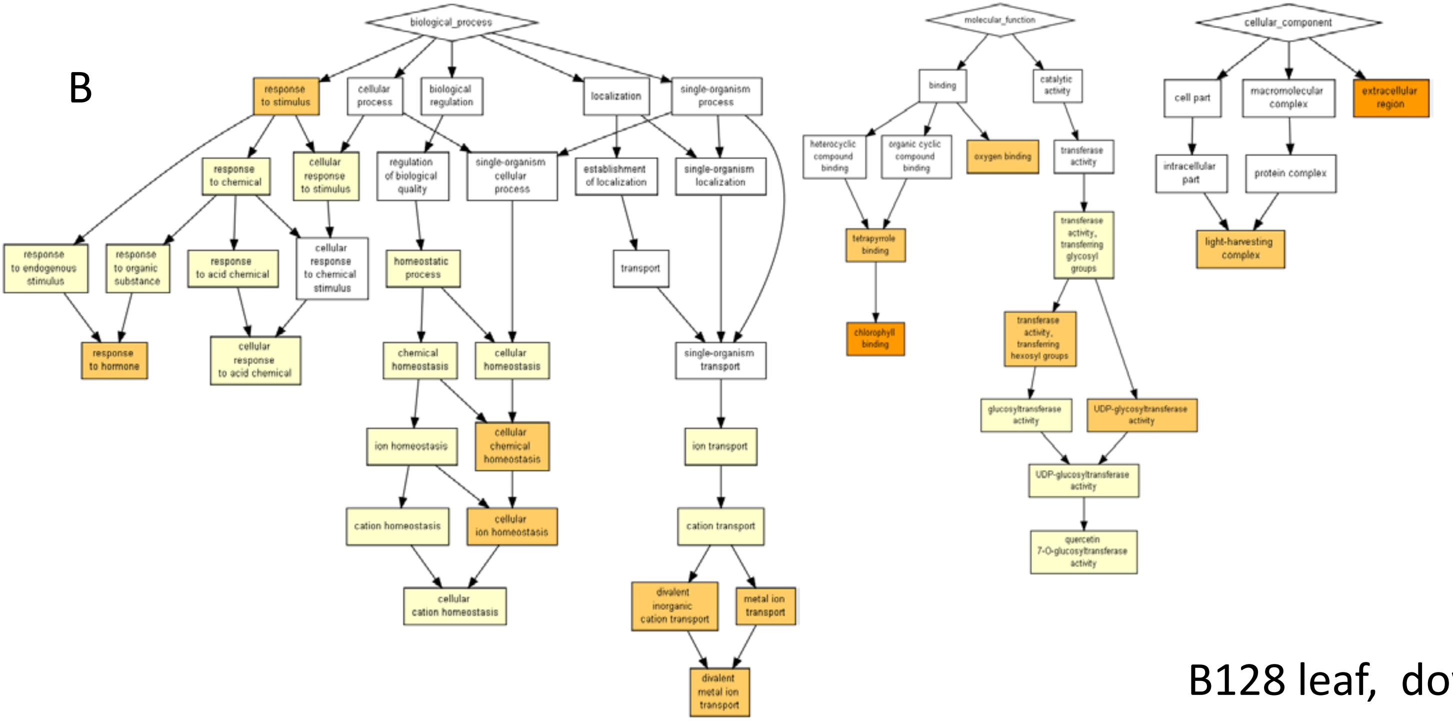

B128 leaf, down-regulated genes

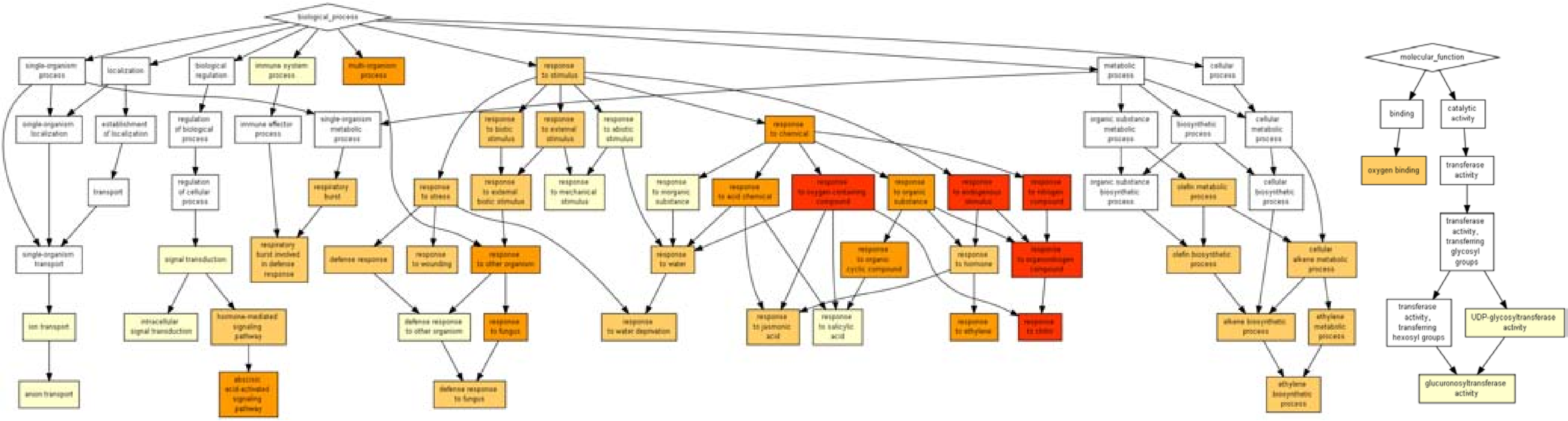

B47 leaf, down-regulated genes

C

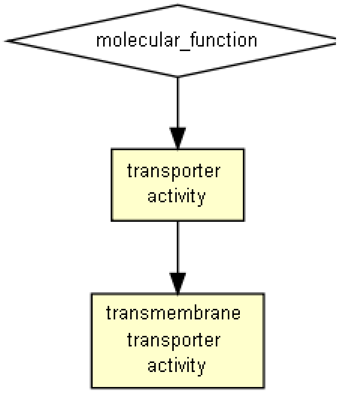

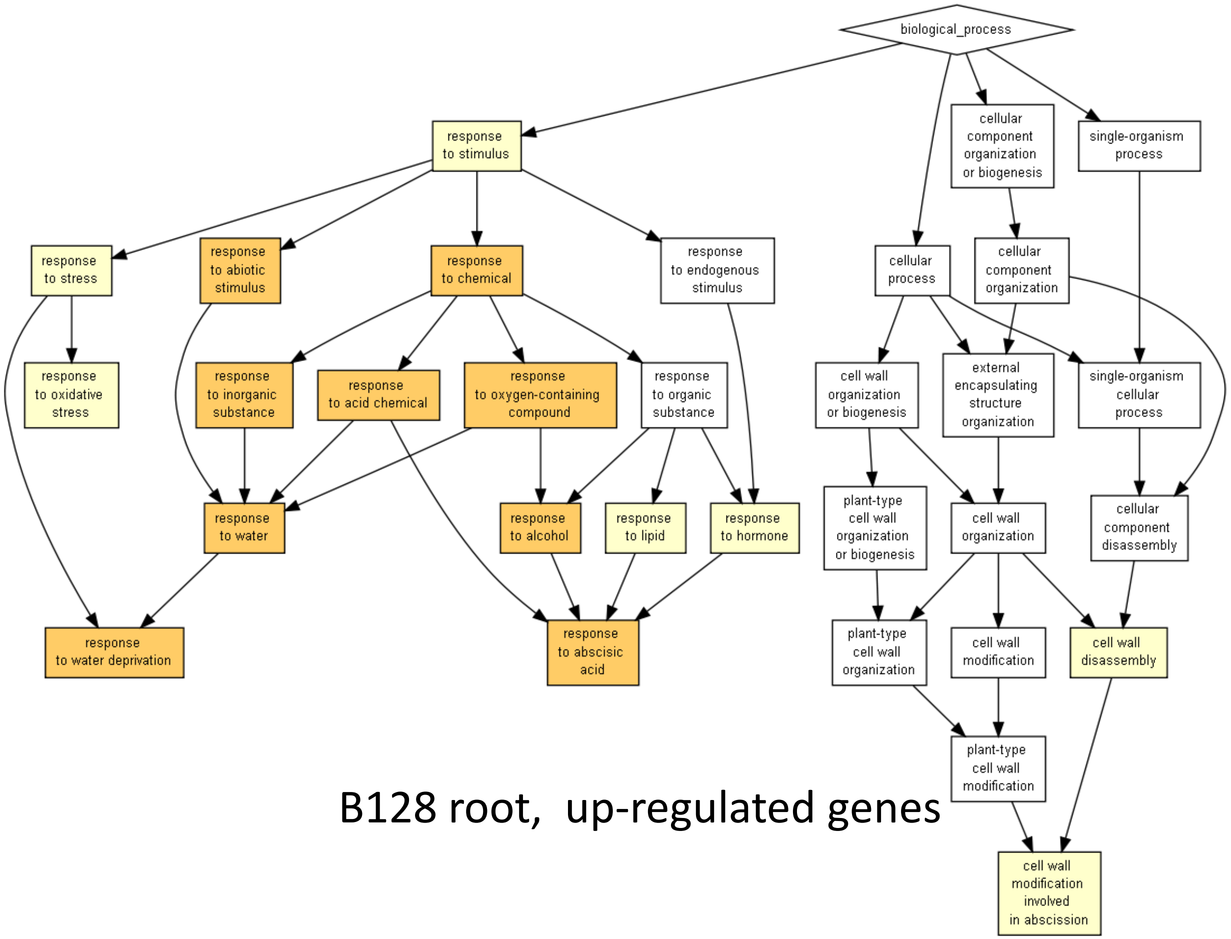

B128 root, up-regulated genes

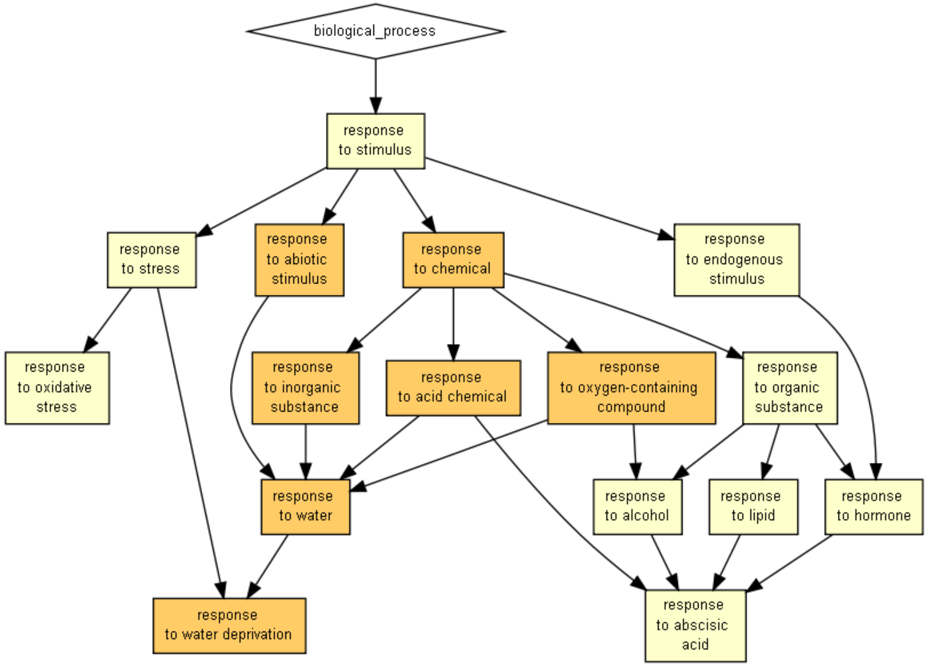

B47 root , up-regulated genes

D

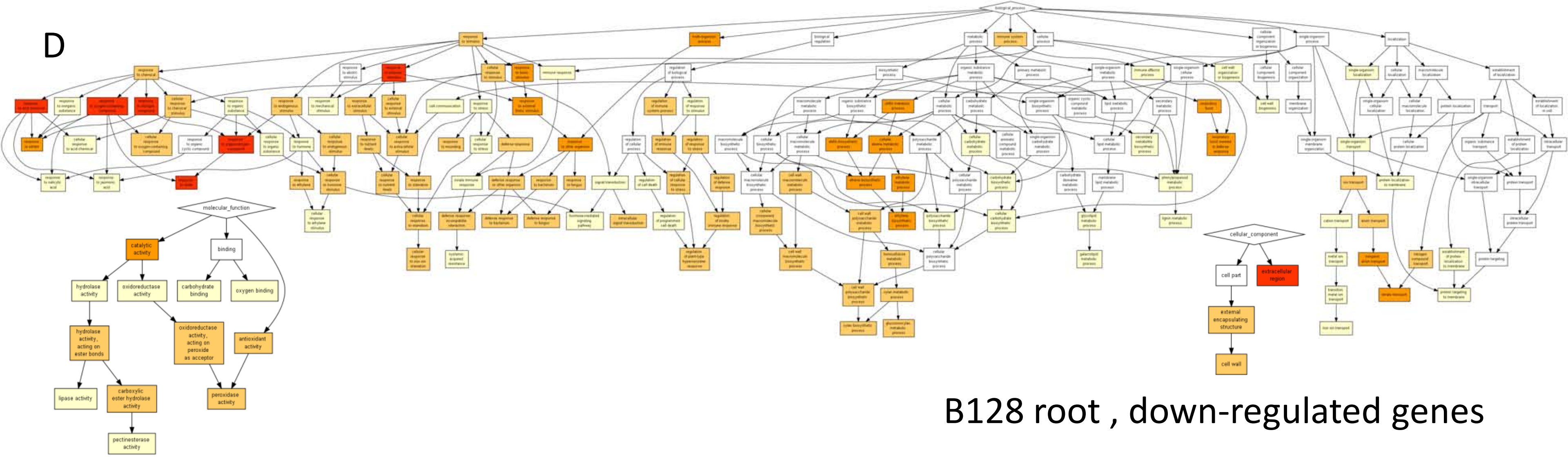

B128 root , down-regulated genes

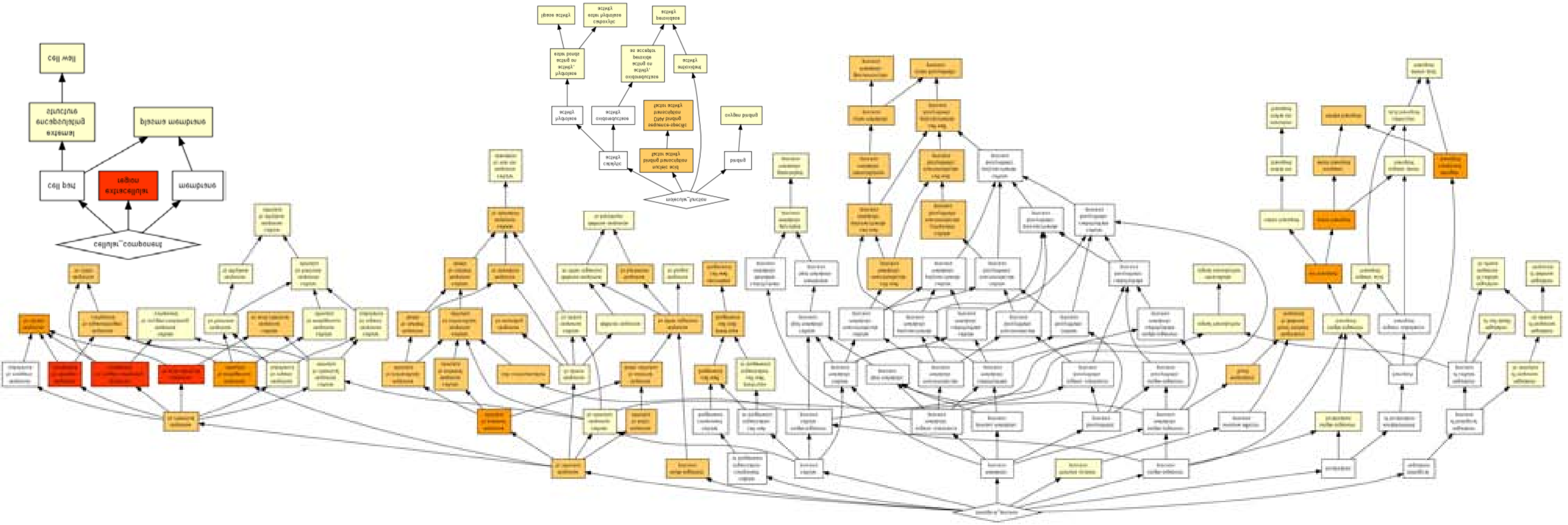

B47 root , down-regulated genes
