# Supplementary figures and images for: Natural variation and gene regulatory basis for the responses of asparagus beans to soil drought
Source: Front Plant Sci. 2015 Oct 27;6:891. doi: 10.3389/fpls.2015.00891 (PMC4621818; doi:10.3389/fpls.2015.00891)

Fig. S2

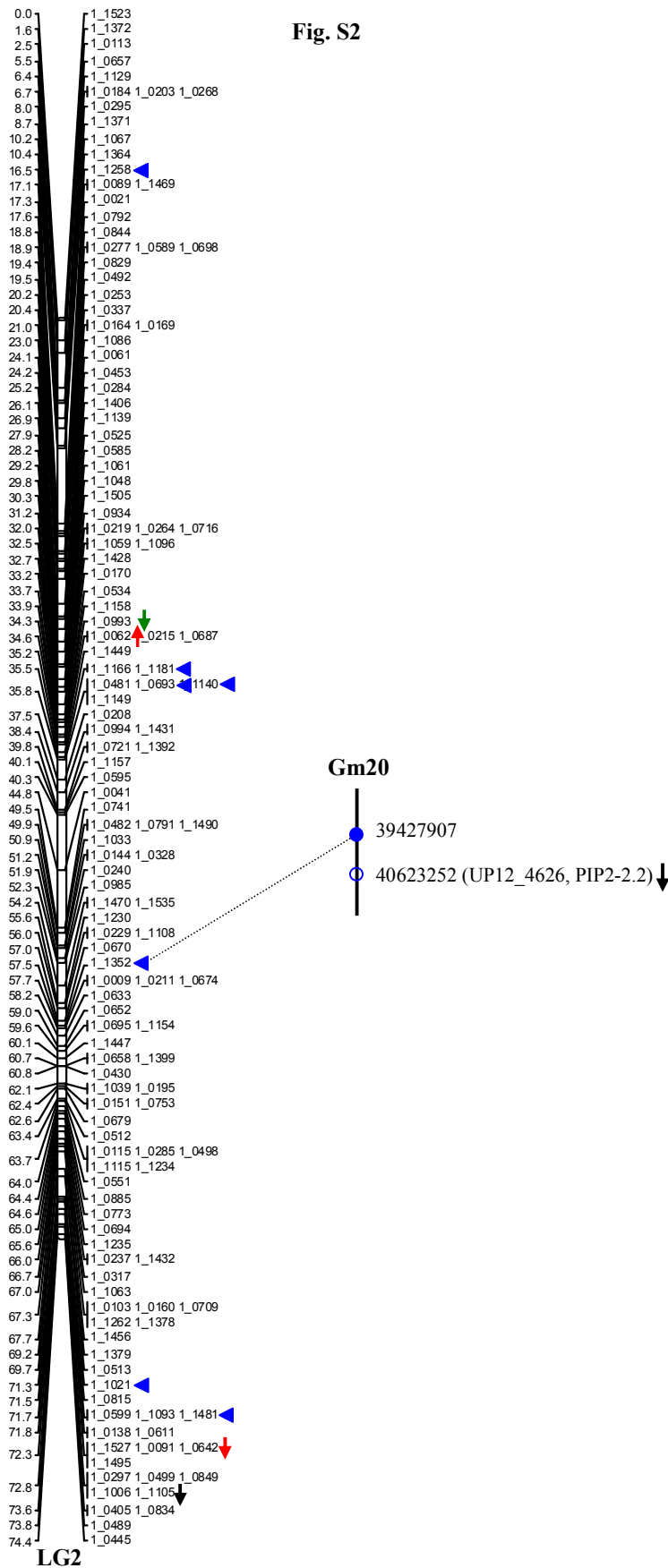

Fig. S4. Cont.

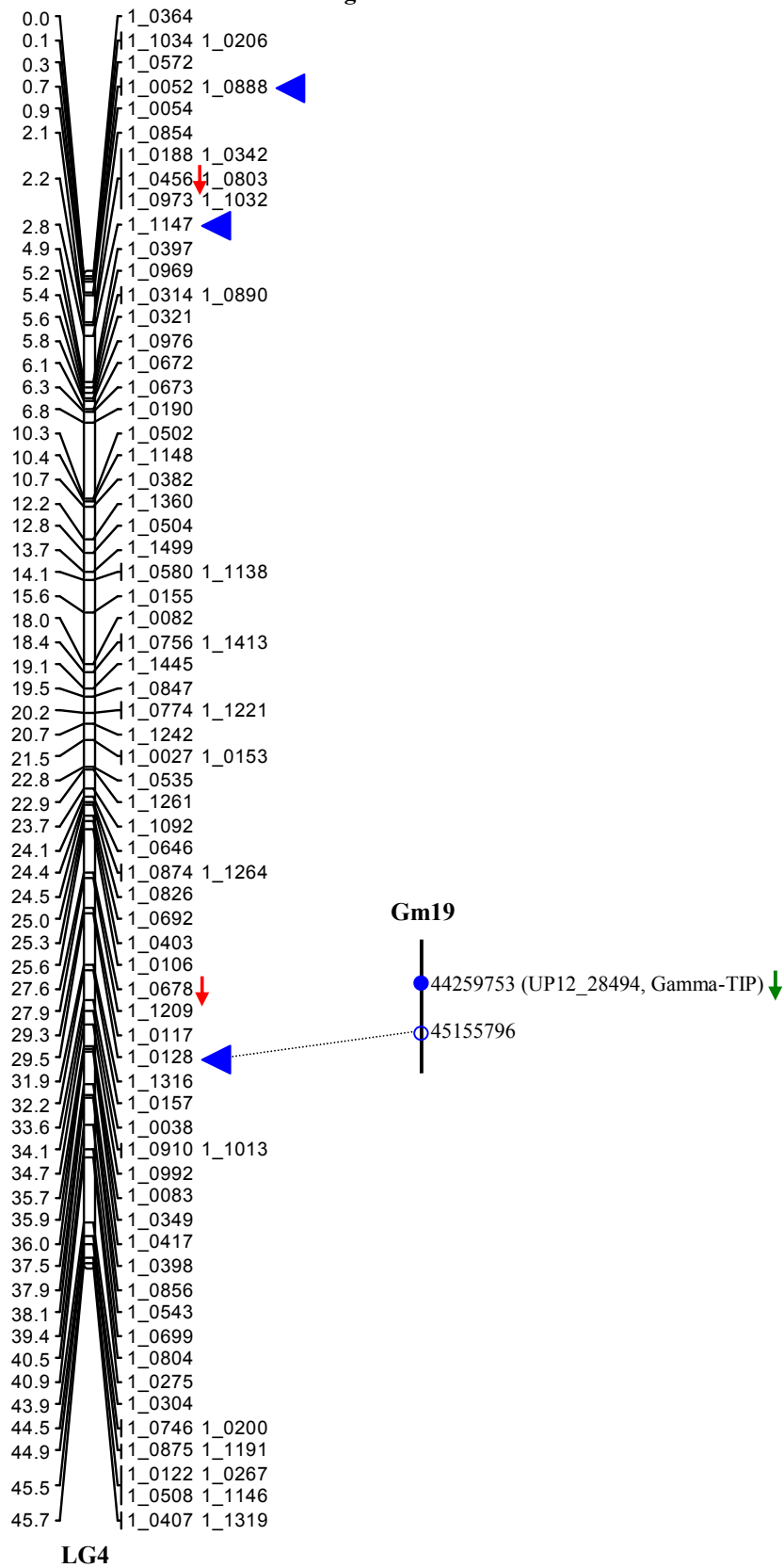

Fig. S4. Cont.

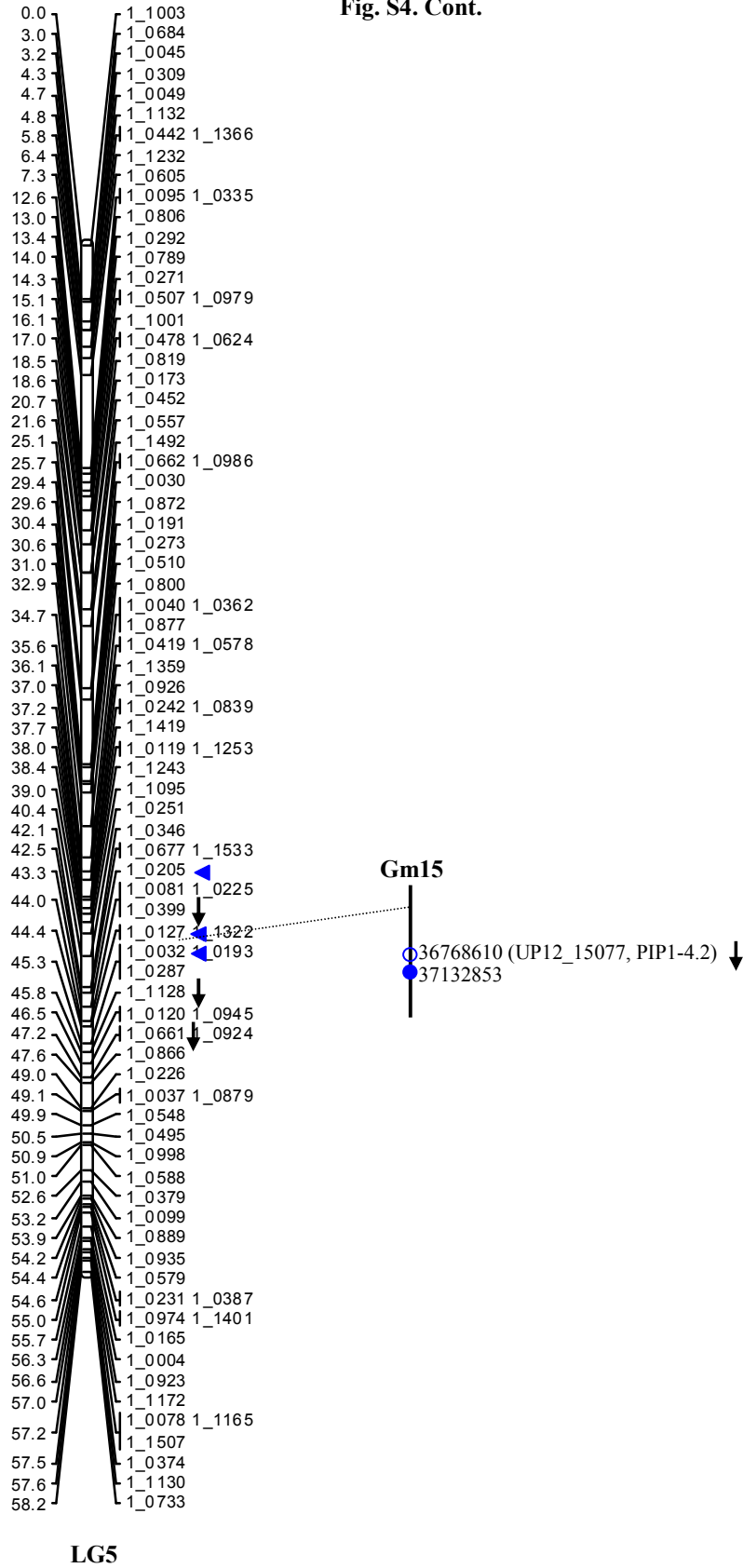

Fig. S4. Cont.

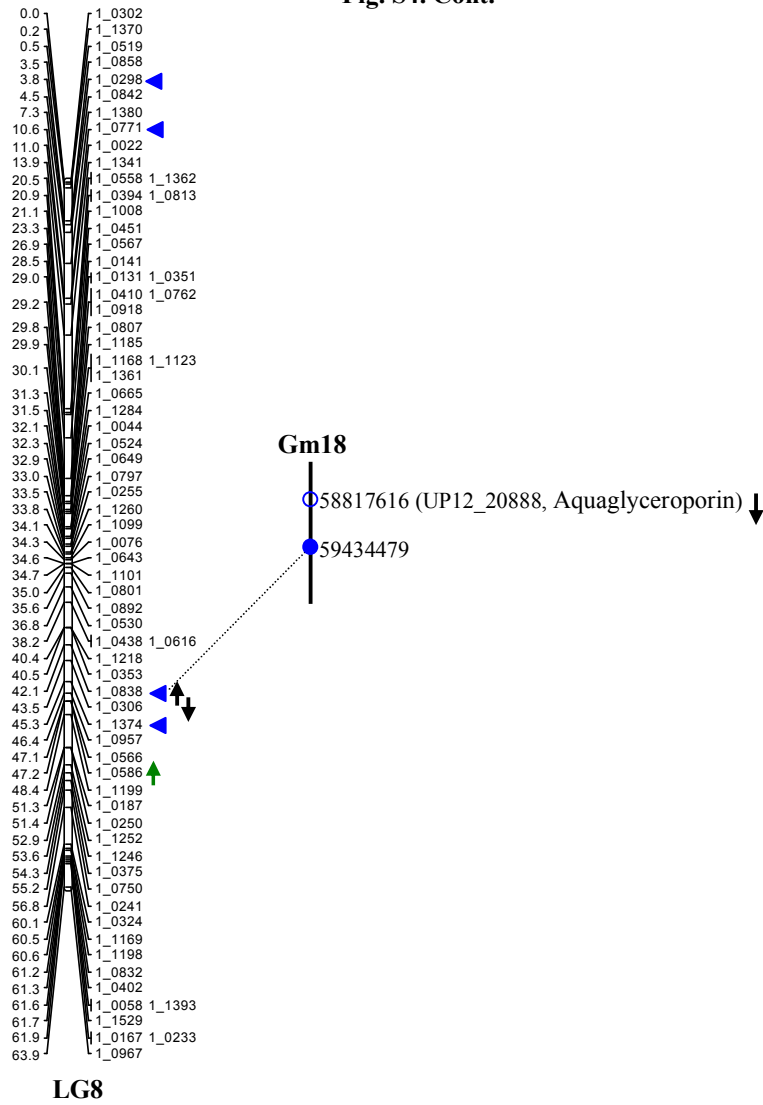

Fig. S4. Cont.

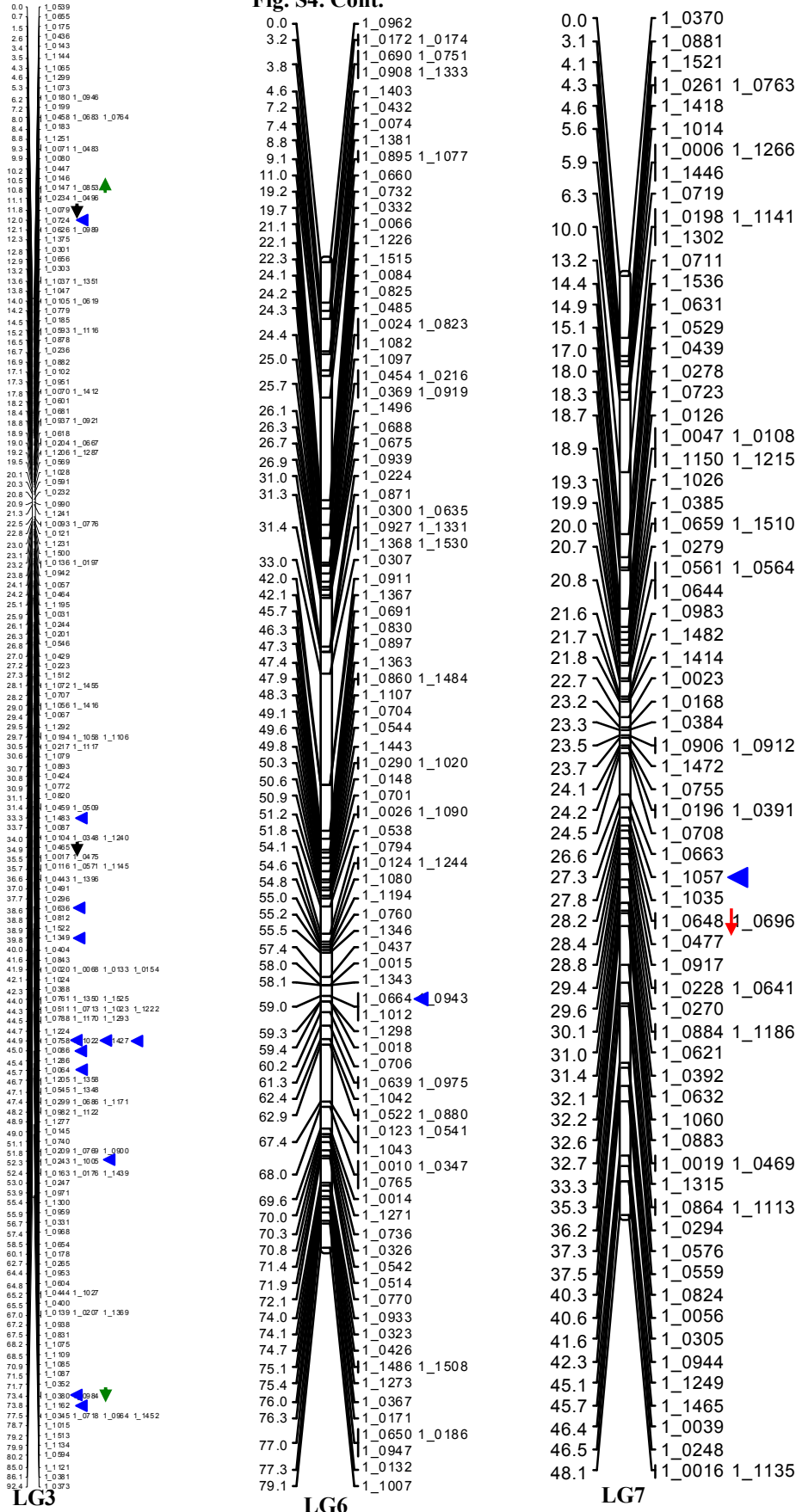

Fig. S4. Cont.

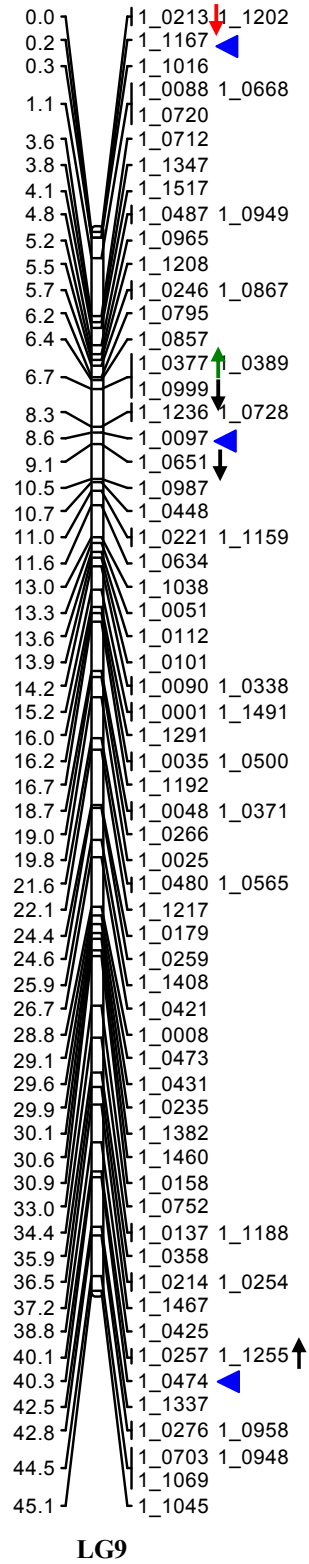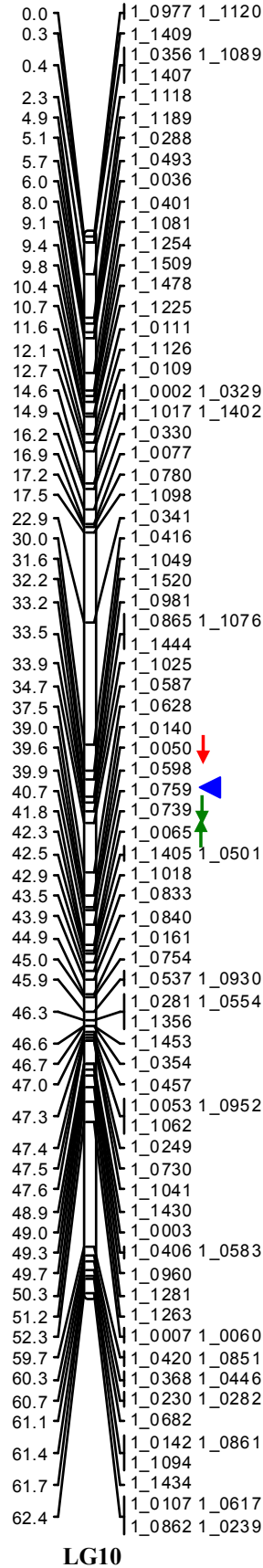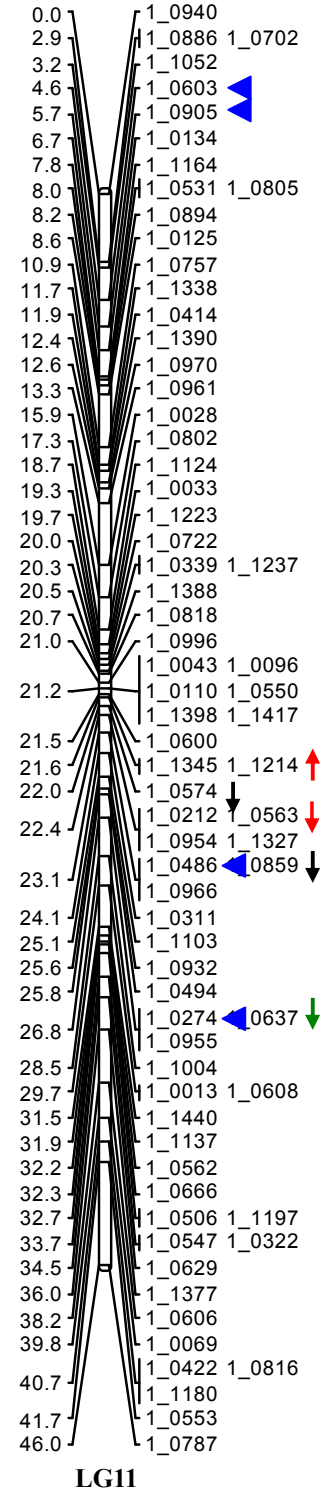

Supplement: Figure S2 — Differentially expressed genes (DEGs) that are in linkage disequilibrium (LD) with the SNPs associated with drought resistance. On the left is the SNP-based genetic linkage map. On the right is the soybean chromosome regions corresponding to the cowpea linkage groups (note that for clarity only orthologous of drAQPs were shown). The loci marked with blue triangles are SNPs significantly associated with drought resistance (P < 0.01). The loci marked with arrows represent DEGs that are in LD with the significant SNPs. Different colors and directions of the arrows indicate the various patterns of transcriptional regulation of the DEGs. Upward arrows: up-regulated DEGs. Downward arrows: up-regulated DEGs. Red arrows: regulated in B47 only. Red arrows: regulated in B128 only. Dark arrows: regulated in both genotypes. [file Image2.PDF]
