# Supplementary material for: Natural variation and gene regulatory basis for the responses of asparagus beans to soil drought
Source: Front Plant Sci. 2015 Oct 27;6:891. doi: 10.3389/fpls.2015.00891 (PMC4621818; doi:10.3389/fpls.2015.00891)
Supplement: Table S1 — Dates of seed sowing and phenotyping in the course of the experiment. [file Table1.DOCX]

**Table S1. Dates of seed sowing and phenotyping in the course of the experiment**

| Actions | Date | |  |
| --- | --- | --- | --- |
|  | 2011 | 2012 | 2014 |
| Seed sowing | Sep 21 | Apr 12 | May 15 |
| Water withholding started | Oct 9 | May 4 | May 27 |
| Scoring Scu | Nov 1 | Jun 1 | June 23 |
| Scoring Wt | Nov 14 | June 13 | June 23 |
| Scoring Stg | Dec 12 | June 16 | June 23 |
